# A single transcriptional regulator is crucial for the adaptation of *Staphylococcus aureus* to diverse niches

**DOI:** 10.1098/rspb.2026.1930

**Published:** 2026-10-14

**Authors:** Claus Vogl, Lynette C. Mikula, Helene Marbach, Katharina Mayer-Weihrauch, Sebastian Herndler, Leonie Breitschopf, Amy C. Pickering, Mary-Elizabeth Jobson, Adriana Cabal Rosel, Nora Dinhopl, Igor Loncaric, Werner Ruppitsch, Lindsey N. Shaw, Orla M. Keane, J. Ross Fitzgerald, Monika Ehling-Schulz, Simon Heilbronner, Tom Grunert

**Affiliations:** ^1^ Unit of Genetics and Animal Breeding, Centre of Biological Sciences, Department for Biological Sciences and Pathobiology, University of Veterinary Medicine, 1210 Vienna, Austria; ^2^ Austrian Agency for Health and Food Safety (AGES), 1220 Vienna, Austria; ^3^ Unit of Microbiology, Center of Pathobiology, Department for Biological Sciences and Pathobiology, University of Veterinary Medicine, 1210 Vienna, Austria; ^4^ The Roslin Institute, University of Edinburgh, Institute of Structural and Molecular Biology, Edinburgh, EH25 9RG, UK; ^5^ Department of Molecular Biosciences, University of South Florida, Tampa, FL 33620-5150, USA; ^6^ Center for Antimicrobial Resistance, University of South Florida, Tampa, FL 33620-5150, USA; ^7^ Unit of Pathology, Center of Pathobiology, Department for Biological Sciences and Pathobiology, University of Veterinary Medicine, 1210 Vienna, Austria; ^8^ Institute of Hygiene and Medical Microbiology, Universität Innsbruck, 6020 Innsbruck, Austria; ^9^ Faculty of Food Technology, Food Safety and Ecology, University of Donja Gorica, 81000 Podgorica, Montenegro; ^10^ Animal & Bioscience Department, Teagasc, Grange, Dunsany, Co. Meath, C15 PW93, Ireland; ^11^ Division of Microbiology, Ludwig-Maximilians-Universität München, 80539 Munich, Germany

**Keywords:** phenotypic plasticity, niche adaptation, host-specific adaptation, *Staphylococcus aureus*, SigB (sigma factor B), bovine mastitis

## Abstract

Adaptation of bacterial pathogens to new hosts often involves the exchange of mobile genetic elements, or generally the highly variable accessory genome. However, adaptation to different niches is often plastic, mediated by conserved pathways in the core genome. The general stress response of most Gram-positive bacteria is a classic case of adaptive plasticity and mediated primarily by the alternative sigma factor B (SigB), a transcriptional regulator. In *Staphylococcus aureus*, however, sigma factor B appears to have been co-opted to regulate the switch between intracellular and extracellular niches. In the milk-rich extracellular niche of the bovine udder, low sigma factor B activity appears advantageous: during persistent bovine mastitis, narrowly adapted strains entirely deficient in sigma factor B have evolved repeatedly from phenotypically plastic sigma factor B-wild-type strains through various mutations. As we show by transfer of both natural bovine isolates and genetically engineered *S. aureus* strains from a complex medium to a milk-rich medium, this genetic assimilation is driven by the cost of phenotypic plasticity. Compared with mutated sigma factor B-deficient strains, plastic strains show long time lags in adapting to milk and slower subsequent growth. Overall, we show how a master regulator in the core genome can drive host-specific niche adaptation.

## 1. Introduction

The Gram-positive bacterium *Staphylococcus aureus* is a commensal and clinically important pathogen that can adapt to various hosts and infect various organs within these hosts. This remarkable adaptability is partly owing to its large genetic variability. Strains can be differentiated according to their core genome and assigned to clonal complexes (CCs), in spite of variability in their accessory genome. CCs are often associated with a specific host [1–3]. When CCs are associated with different hosts, exchange of mobile genetic elements mediates host-specific adaptations so that the accessory genome becomes homogenized between different CCs within the same host. In addition to the adaptive potential of the accessory genome [3,4], transcriptional regulators in the core genome mediate adaptive plasticity that allows *S. aureus* to survive and replicate in both intra- and extracellular niches [5–7]. Sigma factor B (SigB), a member of the sigma factor family of transcriptional regulators, is crucial to this adaptability.

In *S. aureus*, the *sigB* operon contains the genes *rsbU, rsbV, rsbW* and *sigB*. Regulation of SigB activity is complex: RsbW antagonises SigB, RsbV antagonises RsbW and RsbU activates RsbV. Thus, the levels of *sigB* mRNA or SigB protein do not necessarily predict SigB activity; instead, transcription of SigB regulated genes is typically taken as a proxy for SigB activity [8–11]. High SigB activity is known to induce traits necessary for survival and growth of *S. aureus* in the intracellular niche, for example, the production of adhesins, which facilitate invasion of host cells, and of carotenoid pigments, which detoxify the reactive oxygen species within host cells [6,7,12]. By contrast, low SigB activity induces secretion of virulence factors and proteases [13,14], traits necessary for survival and growth in the extracellular niche. SigB thus seems to act as a switch between extra- and intracellular niches in *S. aureus*.

Two of the most common globally distributed bovine-associated CCs are CC97 and CC151 [15]: CC97 strains typically cause only mild, subclinical intramammary infection (IMI) and generally have high adhesion and internalization capacities, indicative of adaptation to the intracellular niche [16–18]. CC151 strains cause more severe inflammation and udder damage, probably owing to the secretion of extracellular virulence factors [19,20]. We postulate that quantitative differences in SigB activity between strains of CC151 and CC97 cause these niche-specific adaptations.

The extracellular niche in the bovine udder can be considered a chemostat: continuous secretion of milk replenishes the nutrients essential for bacterial growth, and milking removes the bacteria and their metabolites. Overall, this creates a relatively stable environment in which the increased secretion of proteinases induced by low SigB activity could benefit *S. aureus* growth and long-term survival. Persistent IMI by *S. aureus* in dairy cows may last for the host's lifetime, allowing for short-term bacterial evolution. In fact, SigB-deficient (SigB-def) strains have been isolated repeatedly from cows with IMI and once the evolution of a SigB-def strain during persistent IMI has been observed [21–23]. We thus propose that bovine SigB-def strains are narrowly adapted to growth in milk and long-term survival in the udder and outcompete the phenotypically plastic SigB wild-type (SigB-wt) strains in this extracellular niche. Furthermore, while human isolates probably have never been exposed to the milk-rich extracellular niche of the bovine udder during their evolution, SigB-def mutations that generally enhance extracellular growth may allow even them to grow in milk.

In this article, we therefore investigate the role of the core gene and master regulator SigB in the adaptation of *S. aureus* to the milk-rich extracellular niche in the bovine udder. We specifically characterize and compare the adaptation of naturally isolated (bovine and human associated) and engineered phenotypically plastic SigB-wt and canalized SigB-def strains to milk-rich media. We show that adaptive plasticity and genetic regulation of SigB activity governs a large part of *S. aureus* niche adaptation.

## 2. Results

### (a) General experimental set-up that enables measuring adaptation to milk

Since the focus of this article is on an ecological shift between complex niches, our experiments involve the transfer of *S. aureus* strains from one complex medium to another, specifically from tryptic soy broth (TSB) to semi-skimmed milk broth (SSM). Generally, *S. aureus* strains pre-cultivated in TSB and then transferred to fresh TSB immediately continue to grow, whereas the same strains transferred from TSB to SSM show an adaptation phase (i.e. a period of slow growth) until they are fully adapted to the new medium and their growth accelerates (figure 1A). We therefore define two target variables, similar to those proposed for monitoring bacterial growth in minimal media [24,25], to assess growth after transfer from one complex medium to another: (i) the duration of the post-transfer adaptation phase (‘adaptation time’ symbolized by τ), which is the initial, possibly multiphasic, slow-growing phase immediately after the strains are moved from TSB to SSM and (ii) the maximum (absolute) growth rate (symbolized by *ρ*_max_) that is reached once the strain is able to optimally utilize the mix of components in the new medium and has therefore (phenotypically) adapted to it (figure 1B; supplementary material, method M1).

**Figure 1.**
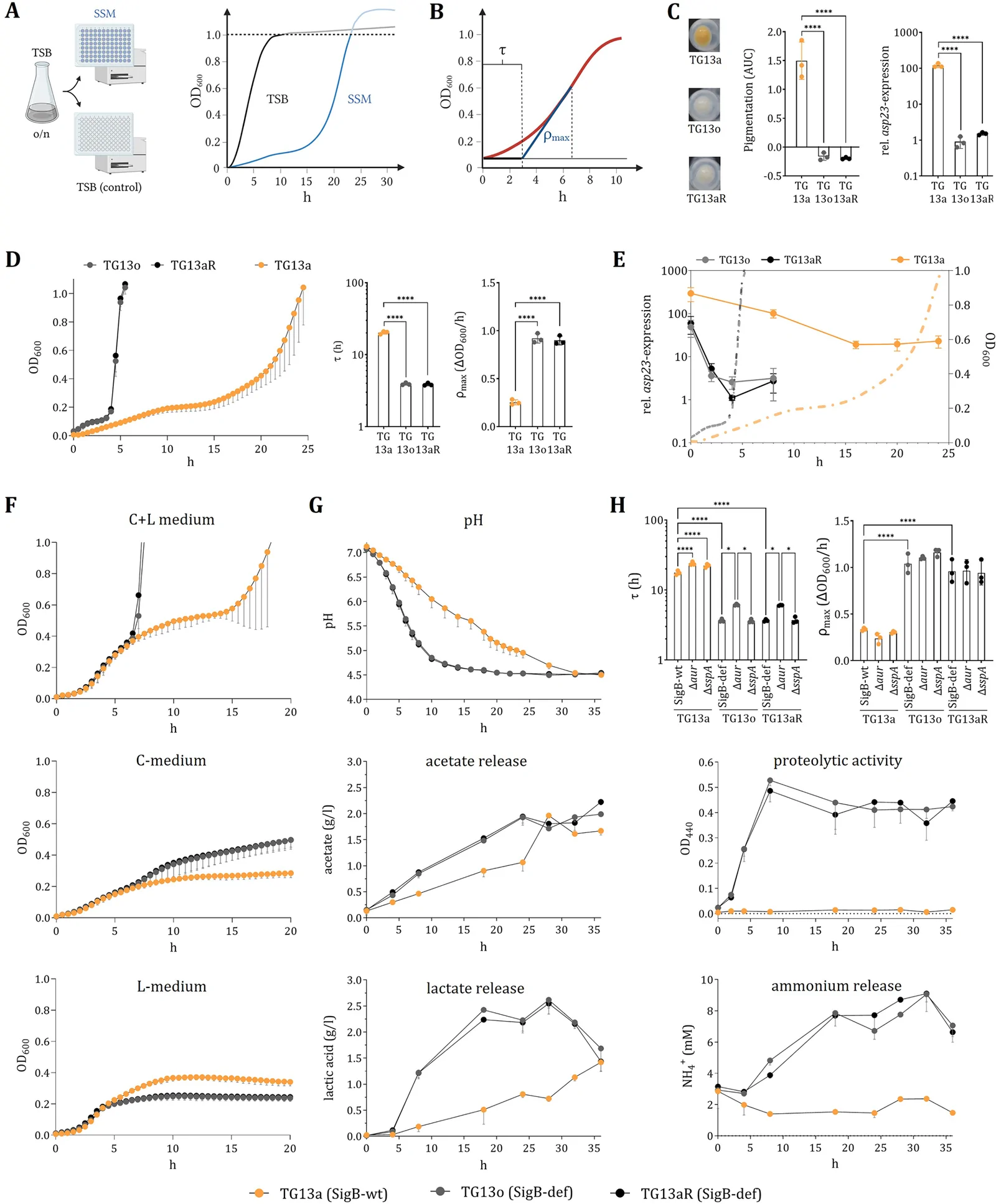
Differences in growth and metabolism between SigB-wt and SigB-def strains. (A) Monitoring of bacterial growth in complex media up to 72 h: after transfer to fresh TSB, *S. aureus* grows immediately; after transfer to SSM, it undergoes an adaptation phase before maximal growth sets in. (B) Calculation of the parameters ‘adaptation time’ and ‘maximum growth rate’ from optical density (OD)_600_ time-series data: from the empirical curve (red), differences of OD_600_ between neighbouring time points are calculated; the maximum difference corresponds to the maximum growth rate *ρ*_max_ (in units of ΔOD_600_/h). The line thus defined is extended to the intersection with the *x*-axis. The length of time from the start of the experiment to this intersection is taken as the adaptation time τ (in units of h). (C–H) Comparisons of TG13a (SigB-wt) and TG13o and TG13aR (both SigB-def) via linear model analyses of untransformed (pigmentation; *ρ*_max_) or log-transformed (reverse transcription quantitative PCR (RT-qPCR), τ) data. Differences in pairwise means were tested with Tukey’s honestly significant difference (Tukey's HSD). (C) Proxies of *S. aureus* SigB activity: (left) carotenoid pigmentation of the bacterial pellet at the bottom of the tube after overnight culture and centrifugation, (centre) measured by absorbance (area under the curve (AUC); mean ± s.d.) and (right) relative *asp23-*mRNA expression (RT-qPCR; mean ± s.d.) in TSB. (D) Growth dynamics in SSM measured at OD_600_ (mean ‒ s.d.). Adaptation time (τ; mean ± s.d.) and maximum growth rate (*ρ*_max_; mean ± s.d.). (E) Simultaneous representation of the changes in relative *asp23*-mRNA expression (RT-qPCR; mean ± s.d.) and bacterial growth (OD_600_) during the adaptation time (TG13a: ≅ 20 h; TG13o and TG13aR: ≅ 4 h). (F) Growth curves at OD_600_ (mean ‒ s.d.) until the end of the experiment or OD_600_ = 1 was reached in media containing casein and lactose (C+L medium), only casein (C-medium) and only lactose (L-medium). (G) Dynamics of acidification and associated metabolites after transfer to SSM up to 36 h later (mean ‒ s.d.): pH, acetate release, lactate release. (H) Contribution of proteases and amino acid metabolism: growth patterns of Δ*aur*/Δ*sspA* isogenic mutants of the SigB-wt (TG13a) and the SigB-def strains (TG13o/TG13aR): maximum growth rate (*ρ*_max_) and adaptation time (τ). Development of proteolytic activity, and ammonium ion (NH_4_^+^) concentration (both mean – s.d.) across the 36 h window. TSB, tryptic soy broth; SSM, semi-skimmed milk broth. Figures were created in BioRender. Grunert, T. (2026) https://BioRender.com/5lskdcp; https://BioRender.com/esdcly5; https://BioRender.com/rngvqnd.

### (b) A sigma factor B-deficient strain that evolved in the mammary gland of its host is better adapted to growth in milk than its sigma factor B-wild-type ancestor

With this experimental setup and analysis approach, we first monitor the growth of SigB-wt and SigB-def *S. aureus* strains collected from a single naturally infected dairy cow during persistent IMI. The SigB-def strain TG13o (CC97) evolved from the original SigB-wt strain TG13a by a point mutation in *rsbU*(G368A) [21], and obviously outcompeted the original isogenic strain over time. The same SigB-def mutation was introduced into SigB-wt TG13a to produce the control strain TG13aR [23] (supplementary material, table S1). When grown in TSB, the carotenoid pigmentation of TG13o and TG13aR (both SigB-def) is low and very similar; that of TG13a (SigB-wt) considerably higher. The same pattern is reflected by the expression levels of the *asp23* gene, which is a well-established proxy of SigB activity [8,9,23] (figure 1C). Overall, these results indicate that the single nucleotide change in the *rsbU* gene causes SigB deficiency. When the strains are pre-cultivated in TSB and transferred to SSM, the adaptation time τ is much longer for the SigB-wt than the SigB-def strains (figure 1D and for TSB control; see supplementary material, figure S1A). Additionally, the SigB-def strains show a higher maximum growth rate *ρ*_max_ than the SigB-wt TG13a (figure 1D). Solid curd forms at the bottom of the sample vials of the SigB-def strains (TG13o and TG13aR) after 5.5 ± 1.2 h (mean ± s.d.) but only after 19.4 ± 1.3 h in vials of the SigB-wt TG13a strain. Counting colony forming units (CFU ml^−1^) confirms that the observed differences in τ and *ρ*_max_ reflect the actual growth pattern and are not driven by clotting or curd formation (supplementary material, figure S1B). Moreover *in vitro* competition experiments confirm that the *in vivo* evolved SigB-def strain (TG13o) outcompetes the original SigB-wt strain (TG13a) in SSM alone, but not in TSB (supplementary material, figure S1C and table S2).

Next, we assess the regulatory adaptation of SigB by monitoring *asp23* expression of the strains during the adaptation and rapid growth phases. Initially, expression of *asp23* is higher in the SigB-wt strain compared with the SigB-def strains (figure 1E; supplementary material, figure S1D). After transfer to SSM, the SigB-def strains show a rapid decrease in *asp23* expression to a low level that is maintained thereafter, whereas the SigB-wt strain shows a gradual decrease to a relatively high level. The lowest level of SigB expression is reached just before or at the time of maximum growth by all strains, however, the absolute times differ: about 8 h for the SigB-def strains (TG13o and TG13aR) and about 24 h for the SigB-wt strain (TG13a).

Overall, the SigB-def mutation causes a short adaptation time after transfer to SSM and a high growth rate, probably allowing the mutant to outcompete the original strain during persistent IMI. Thus, the original adaptive plasticity comes at a cost.

### (c) The sigma factor B-deficient strain has an increased capacity for proteolysis, casein utilization and lactic acid-based fermentation

Casein and lactose are the main protein and main carbohydrate in milk. Strains of varying SigB levels may differ in their ability to metabolize these nutrients. We therefore compare the growth of TG13a, TG13o and TG13aR in synthetic media containing either casein, lactose or both (C-medium, L-medium, C+L-medium, respectively). After pre-cultivation in TSB and transfer to the C+L-medium, both SigB-def strains adapt more quickly and have a higher maximum growth rate than the SigB-wt TG13a isolate. Note that these growth patterns are qualitatively similar to those in SSM (figure 1F). In the media containing only casein (C-medium) and only lactose (L-medium), the maximum density reached by all strains is much lower, with a slight growth advantage for the SigB-def strains (TG13o and TG13aR) in the C-medium and a comparable disadvantage for these strains in the L-medium. Thus, the SigB-def mutant metabolizes casein more efficiently than the SigB-wt in the presence of lactose.

Bovine SigB-wt *S. aureus* strains, such as TG13a, have previously been shown to efficiently utilize lactose and cause acidification indicative of fermentation [3]. We observe a significantly faster decrease in the pH of the SSM media containing the SigB-def strains than in the SSM media containing the SigB-wt strain (figure 1G; supplementary material, table S3), as well as a significantly faster accumulation of acetate and lactate. Interestingly, the SigB-def strains produce mainly lactate as a by-product of fermentation rather than acetate, whereas the SigB-wt strains produce both of these organic acids at relatively low and comparable levels.

SigB-def strains are known to copiously secrete proteases, particularly the zinc metalloprotease aureolysin (Aur) and serine protease glutamyl endopeptidase (V8 protease; SspA) [21,26–28]. These are secreted extracellularly in inactive forms; Aur is capable of not only activating itself but also SspA [29]. To test the connection between the growth patterns of the SigB-wt (TG13a) and the SigB-def (TG13o, TG13aR) strains in milk and their secretion of Aur and SspA, we conduct experiments using isogenic Δ*aur* and Δ*sspA* knockouts of all strains. The maximum growth rate for both isogenic mutants remained unchanged relative to the original strains. However, Δ*aur* and Δ*sspA* interact epistatically with the SigB-def mutation: in the SigB-def strain, the Δ*aur* knockout significantly prolongs the adaptation time to milk, whereas the Δ*sspA* knockout has no effect; in the SigB-wt strain, both knockouts show an increased adaptation time, but not to the degree of the single Δ*aur* knockout in the SigB-def strain (figure 1H). Generally, bacterial protein degradation can also be monitored directly: in milk, casein aggregates primarily in micelles. Using transmission electron microscopy, we detect collapsed casein micelles in the SSM culture media of the SigB-def strain TG13o (supplementary material, figure S1E). We also detect substantial casein degradation in the SSM culture media of the SigB-def isolates TG13o and TG13aR and hardly any protein degradation in the SigB-wt (TG13a) culture media (supplementary material, figure S1F). Correspondingly, proteolytic activity in the culture media of the SigB-def strains increases rapidly within the first 8 h of growth but remains constant at very low levels in that of the SigB-wt strain; ammonia can only be found in the former (figure 1H; supplementary material, table S3).

Overall, the SigB-def mutation induces complex metabolic remodelling that is advantageous for growth in milk: SigB-def facilitates the degradation and utilization of casein and lactose, the main milk components metabolized by *S. aureus*. It further induces metabolic remodelling: lactate rather than acetate becomes the main fermentation by-product of the SigB-def *S. aureus* strains, as in lactic acid bacteria (LAB) [30]. SigB-def strains also show increased excretion of proteases. Knocking out the aureolysin gene has a stronger effect on the adaptation time in SigB-def strains than in the SigB-wt strain.

### (d) In-host evolution towards sigma factor B-deficiency in bovine mastitis isolates of various clonal complexes

Since SigB-def mutations seem to increase fitness in persistent IMI, we screened 105 consecutively sampled isolates of *S. aureus* from 20 bovine hosts for mutations that disrupt the function of SigB (supplementary material, figure S2). We find independent SigB-def mutations in three isolates, of which one is assigned to CC151 and two to CC1; the specific mutations differ between isolates (figure 2A). As controls, we construct isogenic mutants that reverse two of these mutations. In both reconstructed strains, asp23 expression is restored, confirming that the mutations are solely responsible for the inactivation of the *sigB* operon (figure 2B). All three SigB-def strains show qualitatively and quantitatively similar growth patterns to TG13o: a significantly shorter adaptation time after transfer to milk (figure 2C) and a significantly higher growth rate after the initial adaptation phase (figure 2D).

**Figure 2.**
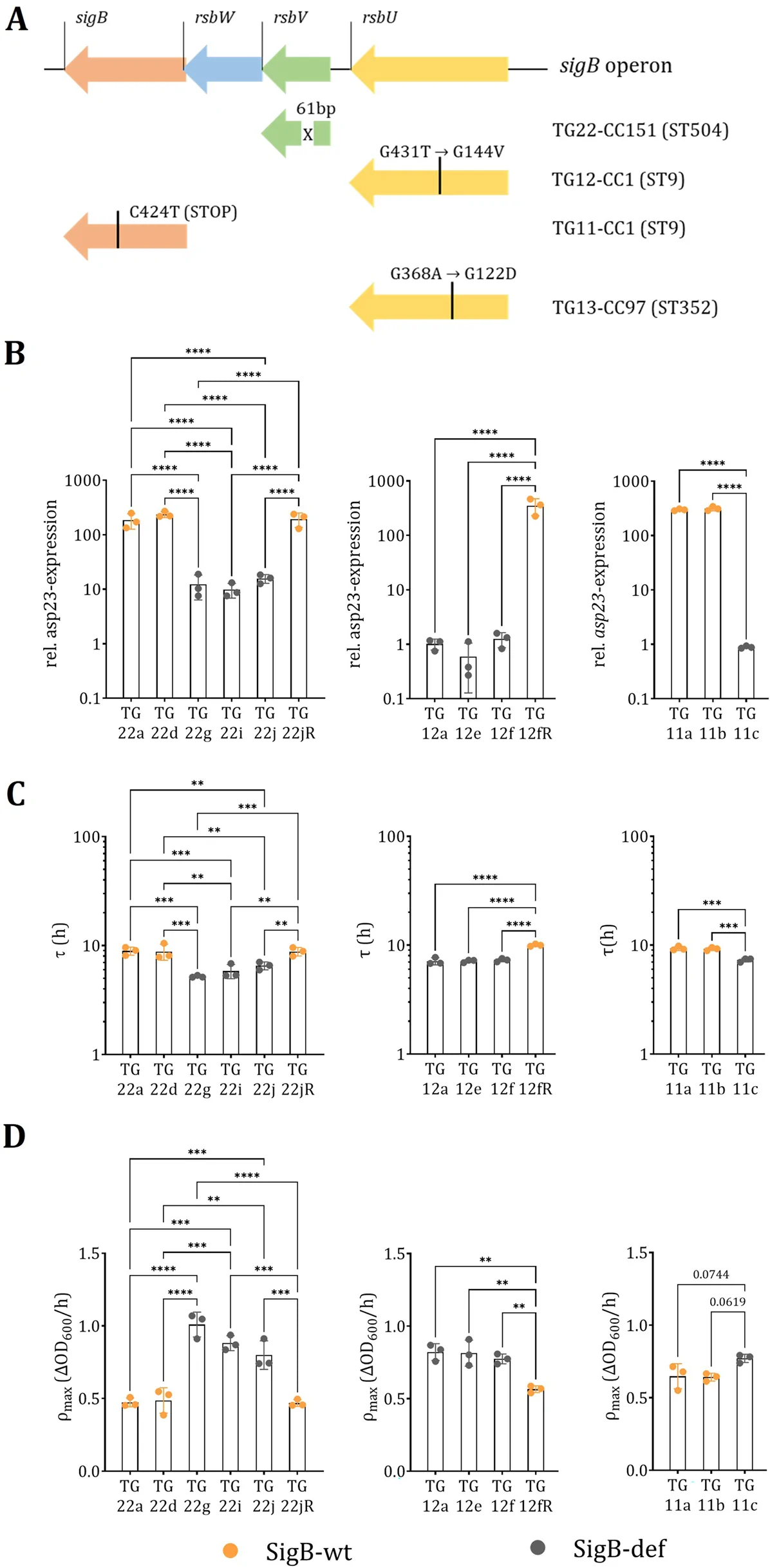
In-host evolution towards SigB-deficiency in bovine mastitis isolates of various clonal complexes. (A) Scheme of the *sigB* operon and mutations causing SigB-deficiency that have been identified in *S. aureus* isolates from persistent IMI in the respective isolates. (B) Relative *asp23-*mRNA expression (RT-qPCR; mean ± s.d.) in TSB. (C) Adaptation time (τ; mean ± s.d.) and (D) maximum growth rate (*ρ*_max_; mean ± s.d.) after transfer to SSM. Differences in pairwise means were tested with Tukey's HSD. SigB-wt: yellow symbols; SigB-def (different mutations): grey symbols.

Overall, this indicates convergent adaptive evolution of *S. aureus* across clonal complexes. Independent mutations in different parts of the *sigB* operon that reduce SigB activity cause a shorter adaptation time after transfer to milk and a subsequent higher growth rate.

### (e) Niche-specific prevalence of the bovine-associated lineages CC97 and CC151 correlates with sigma factor B activity and growth

Recall that SigB-wt strains of CC97 appear well-adapted to the intracellular niche since they have high adhesion and internalization capacities and cause only mild IMI with limited tissue damage [16–18], whereas strains of CC151 often cause severe episodes of IMI and more extensive damage [19,20]. As these differences may be caused by variation in SigB, we examined a geographically and phylogenetically diverse panel of these two CCs (supplementary material, figure S3 and table S4). We use linear models with clonal complex (CC97 vs CC151) as a fixed effect and isolate as a random effect to show that: (i) the CC151 isolates are significantly less pigmented than the CC97 isolates (*p* = 0.0007) and therefore have lower levels of SigB activity, (ii) and that, correspondingly, they have a faster adaptation time and a higher maximum growth rate (both *p*-values < 0.0001) after transfer to SSM. A correlation analysis of all strains, irrespective of their assigned CC, shows a positive relationship between SigB activity (as measured by the level of carotenoid pigmentation) and the adaptation time to SSM, as well as a negative relationship between SigB activity and the maximum growth rate (figure 3A).

**Figure 3.**
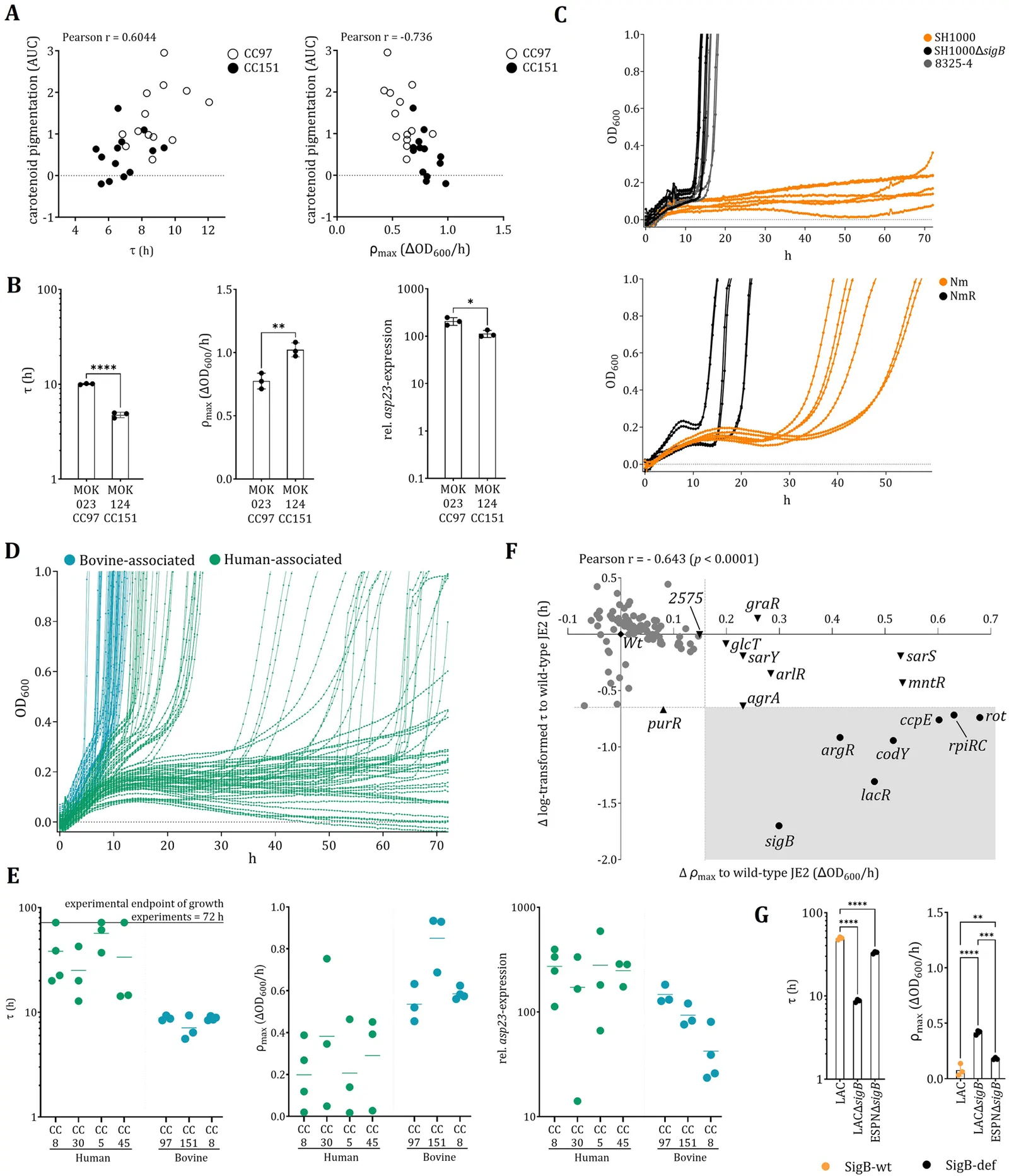
Variation of SigB activity and growth in milk among bovine- and human-associated isolates and screening of NTML regulatory mutant strains. (A, B) Among bovine-associated isolates of CC97 and CC151. (A) Carotenoid pigmentation (AUC) positively correlates with the adaptation time (τ), and negatively correlates with the maximum growth rate (*ρ*_max_) of CC151 (*n* = 13) and CC97 (*n* = 13) isolates. Each point represents one isolate of CC151 (black) and CC97 (white). (B) Comparison of MOK023-CC97 and MOK124-CC151 after transfer to SSM. Differences in means were evaluated using *t*-tests of log-transformed data for relative *asp23*-mRNA expression and for adaptation time (τ) and of untransformed data for maximum growth rate (*ρ*_max_), respectively. (C) Among human-associated SigB-wt and SigB-def strains. After transfer to SSM, human-associated SigB-wt strains grow late and slowly while the isogenic SigB-def strains grow faster. Shown here are the human-derived SigB-def strain 8325-4 (grey), the repaired SigB-wt mutant SH1000 (orange) and the engineered SigB-def mutant SH1000Δ*sigB* (black) as well as the human-derived Newman strain Nm (SigB-wt, orange) and the isogenic SigB-def mutant NmR (black). (D, E) Among human- and bovine-associated isolates. (D) Bovine-associated strains (blue) grow faster than human-associated strains (green), irrespective of the CC. (E) Adaptation time (τ) and maximum growth rate (*ρ*_max_) after transfer to SSM. The adaptation time was set to 72 h for three human isolates because they did not reach an OD = 0.45 after 72 h or did not grow at all. SigB activity determined by relative *asp23*-mRNA expression in TSB. (F) Screening of Nebraska Transposon Mutant Library (NTML) regulatory mutant strains. Scatter plot and correlation of the differences in the log-transformed adaptation time (τ) and in the maximum growth rate (*ρ*_max_) between the 103 NTML regulatory mutant strains and the original JE2 strain. Seven mutants exhibiting a significantly shorter adaptation time as well as a significantly higher maximum growth rate than the JE2 strain are underlaid with a grey rectangle. (G) Impact of protease-null mutants on *S. aureus* growth in a SigB-def background on the adaptation time (τ) and the maximum growth rate (*ρ*_max_). An ANOVA on log-transformed data was performed for adaptation time (τ) and on untransformed data for the maximum growth rate (*ρ*_max_); in both cases, differences in means were tested with Tukey's HSD.

We corroborate these results by separately analysing a CC97/CC151 strain pair that has been clinically well-characterized *in vitro* and *in vivo* via infection experiments [17,18,20]. *In vivo*, the strain MOK023-CC97 causes mild mastitis, whereas the strain MOK124-CC151 causes severe clinical disease [20]. These two strains have been shown to differ in growth kinetics within the udder: MOK124-CC151 reaches a significantly higher bacterial load than MOK023-CC97, with CFU ml^-1^ peaking at 24 h post-infection (hpi) [20]. Milk clotting follows the same pattern, appearing earlier and more intensely with MOK124-CC151 (first detected at 31 hpi) than with MOK023-CC97 (mild and not apparent until 79 hpi) [20]. The *in vitro* growth phenotypes for MOK023-CC97 and MOK124-CC151 strains in SSM mimic those observed *in vivo* immediately after onset of the bovine intramammary challenge experiment [20]: MOK124-CC151 has a significantly shorter adaptation time than MOK23-CC97 and a significantly higher maximum growth rate. Note that both strains are SigB-wt but differ significantly in their *asp23* transcription levels, with MOK124-CC151 specifically showing reduced SigB activity (figure 3B).

Overall, differences in SigB activity between SigB-wt strains of different CCs allow them to more effectively adapt to and subsequently survive in specific niches of the bovine udder.

### (f) Human-associated *S. aureus* strains with sigma factor B-deficient mutations adapt better to milk than otherwise identical sigma factor B-wild-type strains

If low SigB activity generally enhances growth in milk, then human-derived strains—the major source of newly emerged bovine-adapted strains [15,31]—could similarly exhibit enhanced growth if they are SigB deficient. Indeed, human-associated SigB-def strains of the globally, well-distributed CC8 lineage [32] (both isolated and engineered) grow vigorously after about 15 h while the otherwise genetically identical SigB-wt strains grow only much later and more slowly if at all (figure 3C; supplementary material, table S5). Note that the adaptation times for the SigB-def human-isolated strains in figure 3C are actually long compared with even the SigB-wt bovine strains in figure 3D. This is expected since human-isolated strains may not have encountered a milk-rich niche during their evolution.

Overall, human-derived strains that grow slowly or not at all in milk can adapt to growth in milk through deletion of SigB.

### (g) Bovine-associated *S. aureus* strains grow much better in milk than human-associated strains

We now extend our analysis of the growth patterns of human SigB-wt isolates by including strains from CC5, CC30 and CC45 in addition to strains from CC8 [32], and can confirm that they all grow slowly in milk if at all (figure 3D; supplementary material, table S6). By comparison, the bovine *S. aureus* strains assigned to CC8, CC97 and CC151 all grow well when transferred to SSM (see figure 3E for adaptation time: *p* = 0.0320 and for maximum growth rate: *p* = 0.0382). Using linear mixed models with strain and CC as additional regressors, where we excluded the bovine-associated CC8 strain because it only recently host-jumped to cows [33,34], we find that while expression of *asp23* is lower on average in the bovine strains, the difference is not significant (*p* = 0.2308; figure 3E). This indicates that SigB activity is not the sole driver of the increased growth rates of bovine strains and additional metabolic adaptations seem to be involved. However, when CC8 is considered separately, we observe lower SigB activity in the bovine strains (*p* = 0.0004) that matches their faster adaptation time (*p* < 0.0001) and higher maximum growth rate (*p* = 0.0005).

Overall, SigB activity does not differ between human and bovine strains or CCs, although the bovine strains adapt better to milk. However, in the case of CC8, where human and bovine isolates are particularly closely related, we observe lower SigB activity levels in the bovine strains that matches their faster growth in milk. Therefore, modulation of SigB activity may initiate a broader metabolic remodelling process.

### (h) A transposon mutant library screen shows that the *sigma factor B* operon is the staphylococcal transcriptional regulator with the shortest adaptation time to milk

Such a metabolic remodelling process could be driven by a diverse set of transcription factors. We therefore analyse the growth patterns and proteolytic activity of a subset of the Nebraska Transposon Mutant Library (NTML) [35], comprising every available mutant with transposon insertions in transcriptional regulator genes, that is, sigma factors and transcription factors (*n* = 103; supplementary material, table S7) [36]. The original JE2 *S. aureus* strain that forms the reference of the library was derived from a human CC8 isolate; as expected, JE2 adapts and grows relatively slowly after transfer to SSM. The *sigB* mutant has the shortest adaptation time of all mutant strains and a comparatively high maximum growth rate (supplementary material, figure S4A,B; figure 3F).

Although casein is the most important source for amino acids in milk and can be efficiently utilized by SigB-def strains, the proteolytic activity of *S. aureus* strains surprisingly does not appear to influence their adaptation time or growth rate in this screen (supplementary material, figure S4C,D). While loss of SigB activity induces faster growth in SSM, the growth phenotype is only partially reverted to the wild-type level when all staphylococcal proteases are absent (∆*sigB*∆*aur*∆*sspAB*∆*scpA*∆*spl*; figure 3G). Thus, the loss of SigB causes further not yet fully investigated metabolic remodelling that is favourable for growth in milk, independent of increasing the proteolytic activity. Similar remodelling is induced by knockouts of the negative regulator of the lactose utilization regulator *lacR*, of the regulator of the branched-chain amino acid synthesis *codY* and of the regulator of arginine biosynthesis *argR*, which are among the seven mutants with significantly decreased adaptation times and increased growth rates.

Overall, low SigB-activity seems to ‘pre-adapt’ even human-associated strains so that they grow well in milk. This is evidenced by the *sigB* mutant having the shortest adaptation time of all mutants that disrupt the functions of the transcriptional regulator genes of *S. aureus*, as well as a comparatively high maximum growth rate. *S. aureus* proteolytic activity only partially contributes to the elevated growth phenotype in milk, alongside significant metabolic remodelling in SigB-def strains.

## 3. Discussion

In this article, we investigate the role of the core gene and master regulator SigB in the adaptation of *S. aureus* to the milk-rich extracellular niche in the bovine udder. We model the *in vivo* growth behaviour of *S. aureus* in the milk-rich extracellular niche with an *in vitro* system where strains of *S. aureus* that have been maintained in the standard laboratory medium TSB are switched to semi-skimmed milk broth (SSM) and their adaptation time and maximum growth rate in SSM are subsequently monitored. With this approach, we show that the phenotypically plastic SigB-wt strain TG13a, isolated early during a persistent IMI and assigned to CC97, can adapt well to milk. However, the SigB-def mutant strain TG13o, which evolved later during IMI and completely replaced its ancestor, has an even shorter adaptation time and a higher growth rate in milk. Genetic engineering confirms that the SigB-def mutation causes this growth advantage and is associated with broader metabolic remodelling: SigB-def leads to reduced expression of traits facilitating adaptation to the intracellular niche (carotenoid pigmentation and expression of adhesins) and to increased expression of traits facilitating adaptation to the extracellular niche (degradation and utilization of the main components of milk, casein and lactose). In a competition experiment, the SigB-def strain outgrows the SigB-wt strain after a switch from TSB to SSM, whereas neither strain has a consistent advantage in TSB. Other *S. aureus* strains isolated from persistent IMI have evolved repeatedly from SigB-wt towards SigB-def with similar phenotypic consequences, irrespective of their CC. The evolution of SigB-def strains with canalized phenotypes narrowly adapted to milk from SigB-wt strains with plastic phenotypes capable of expressing carotenoid pigments and adhesins and therefore exploiting an intracellular niche [6,7,12] constitutes a case of genetic assimilation. This genetic assimilation appears driven by the cost of phenotypic plasticity: longer time-lags in adapting to milk and subsequent slow growth [12,37]. The two main bovine clonal complexes of *S. aureus* are CC97 and CC151. SigB-wt strains of CC97 cause subclinical IMI and seem to be particularly well-adapted to the intracellular niche, whereas SigB-wt strains of CC151 cause episodes of clinical IMI and seem better adapted to the extracellular niche [16–20]. These differences in clinical severity and niche specificity are consistent with the results obtained from comparison of SigB-def mutant strains with their SigB-wt isogenic progenitors in our *in vitro* system: lower levels of SigB activity in CC151 compared with CC97 correlate with shorter adaptation times and faster growth. Thus SigB activity seems to both quantitatively and qualitatively regulate the niche use of bovine-adapted strains: subtle shifts in the phenotypically plastic strains are quantitative changes, and loss-of-function mutations to a canalized phenotype are qualitative changes (figure 4). Notwithstanding the adaptive potential of mobile genetic elements [3,4], adaptive plasticity and evolution mediated by SigB, a core gene and master regulator of transcription, is crucial for niche adaptation in *S. aureus*. Note that our *in vitro* system cannot fully recapitulate the intramammary ecosystem, in particular the intricate and dynamic interactions between host immunity and bacterial defences. An experimental set-up beyond the scope of this study would be required to more precisely model intracellular growth and persistence *in vitro*.

**Figure 4.**
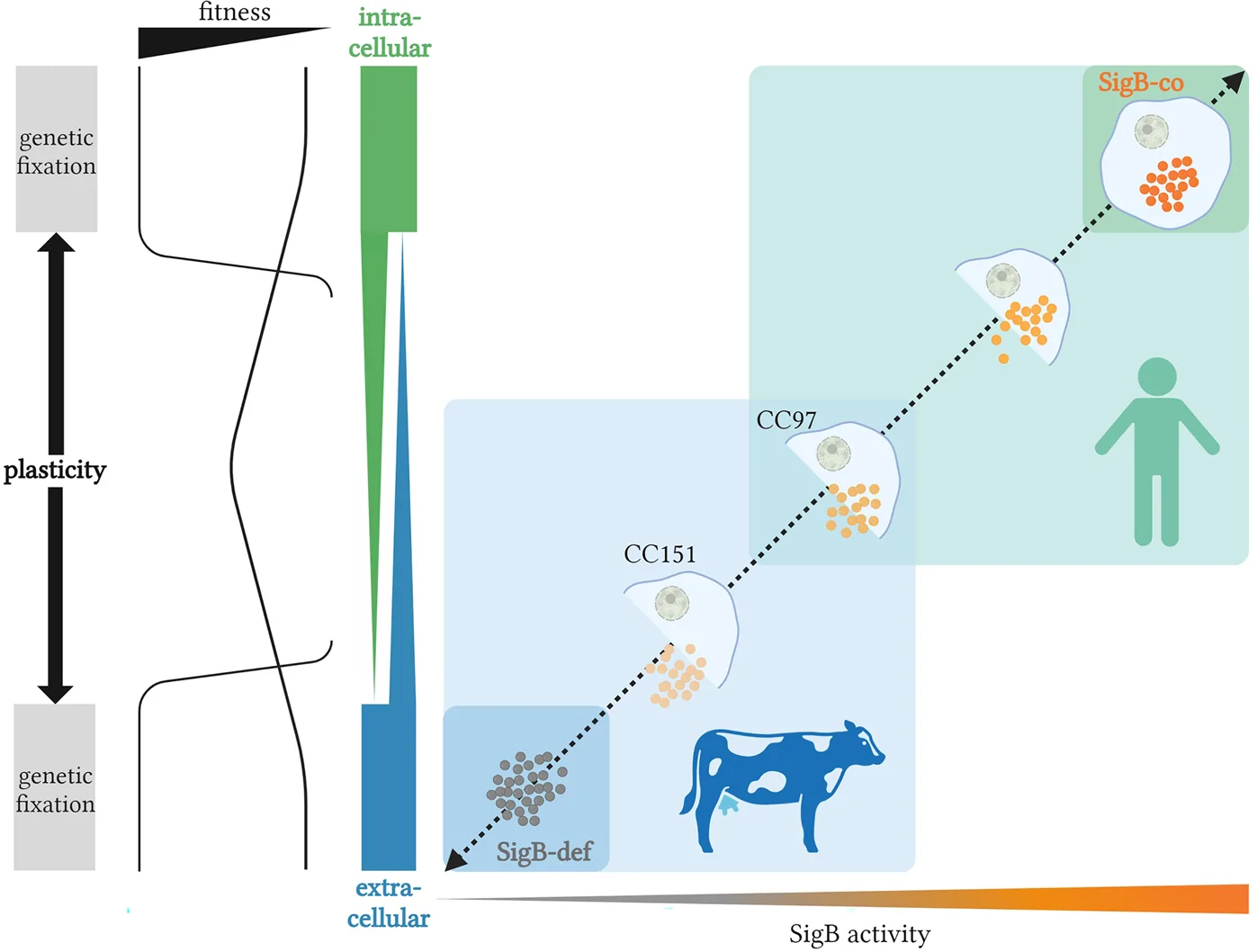
Plastic and genetic regulation of SigB activity governs a large part of *S. aureus* niche adaptation. Model of the divergent roles of SigB in the adaptation of *S. aureus* to intra- and extracellular niches showing the connections between the SigB genotype and carotenoid pigmentation (a proxy of SigB activity and a known adaptation to the intracellular niche). High SigB activity induces traits necessary for intracellular survival and growth; low SigB activity induces traits for extracellular growth. The ability to switch between niches seems to require a plastic SigB-wt state. At both ends of the spectrum, SigB-wt strains are outcompeted by SigB-def or SigB-co strains. SigB-wt bovine isolates of CC97 generally exhibit relatively high levels of SigB and have lower fitness in milk than SigB-wt bovine isolates of CC151. Overall, bovine-associated isolates adapt more readily to milk than human-associated isolates. Created in BioRender. Grunert, T. (2026) https://BioRender.com/a56j023.

SigB-wt strains isolated from humans have probably never encountered a milk-rich extracellular niche, such as the bovine udder during their evolution. As expected, they generally grow slowly when switched to milk, if at all. Nevertheless, the isogenic SigB-def counterparts of these human-associated SigB-wt strains adapt relatively quickly to milk and then grow well. Thus, the SigB-def mutation seems to ‘pre-adapt’ [38] human strains to a milk-rich environment. Indeed, SigB-def mutants in *S. aureus* may facilitate adaptation to extracellular niches in general rather than to only milk-rich ones: clinical isolates from persistent human cystic fibrosis (CF) infections are often characterized by SigB-def mutations [39–42]. Conversely, passaging a SigB-wt strain repeatedly in the intracellular niche of cultivated macrophages produced a hyperpigmented phenotype that resulted from a mutation that canalized (i.e. fixed) constitutive overexpression of SigB (SigB-co) [37]. SigB-def and SigB-co strains thus seem narrowly adapted to the extracellular or intracellular niche, respectively, whereas phenotypically plastic SigB-wt strains seem able to switch between these niches, at the cost of not being optimally adapted to either niche.

While SigB orchestrates a general stress response in ancestral saprophytic relatives, for example, *Bacillus subtilis* [43], *S. aureus* lacks homologues to some genes in the SigB-dependent general stress pathway of *Bacillus subtilis*: the stressosome proteins RsbR, RsbS and RsbT and the energy stress-sensing proteins RsbP and RsbQ [43,44]. In *S. aureus*, the transcriptional regulator SigB thus seems to have been co-opted from its ancestral function in the regulation of the stress response to a derived function as a switch between extra- and intracellular niches. As in *S. aureus*, modulation of SigB activity is crucial for the transition between intra- and extracellular niches in *Listeria monocytogenes* [45]. By contrast to *S. aureus*, however, expression levels of SigB are high in the extracellular host niche and low in the intracellular niche [46]. This reversal of the regulatory mechanism of SigB (low vs high in the extracellular niche) indicates that its convergent role in niche adaptation evolved independently in both lineages. A search in OrthoDB showed that Listeria still possesses RsbR, RsbT and RsbQ but, like *S. aureus*, has lost RsbP, and thus appears intermediate between the ancestral state of *Bacillus* and the derived state of *Staphylococcus*.

Importantly, remodelling of metabolic processes that are not regulated by SigB also appear to contribute to host-specific adaptation: although there is no significant difference in the average SigB activity of bovine and human SigB-wt isolates (as measured via proxies of SigB activity), all bovine isolates adapt better to milk than human isolates. Screening a transposon mutant library, we identify the regulators of some of these metabolic processes: the negative regulator of lactose utilization LacR, as well as the regulators of the branched-chain amino acid synthesis CodY and arginine biosynthesis ArgR. Their pathways have previously been identified as important for growth in milk using a multi-omics approach [47].

The evolution of the canalized SigB-def mutations from the plastic SigB-wt constitutes a case of genetic assimilation. Analogously, persistent intracellular infections may lead to fixation of canalized SigB-co mutations, since long-term intracellular passage of *S. aureus* appears to favour high SigB activity that may be genetically fixed [12,37]. Thus, canalization of high or low SigB activity levels when *S. aureus* persists long enough in exclusively intra- or extracellular niches may be a general phenomenon. However, phenotypic plasticity and therefore the ability to switch between intra- and extracellular niches may be necessary to infect new hosts. Instead of qualitative changes via SigB-def or SigB-co mutations, maintenance of adaptive plasticity and quantitative, polygenic modulation of SigB activity [45] may ensure long-term survival and continued adaptability.

## 4. Material and methods

### (a) Strains

All strains were stored in glycerol stocks at −80°C and were pre-cultivated on tryptic soy agar (TSA; Thermo Fisher Scientific, Oxoid, Hampshire, UK) at 37°C for 24 h and kept at 4°C for a maximum of 2 weeks until use. TSA for strains of the Nebraska Transposon Mutant Library (NTML) and the SH1000∆*sigB* mutant was supplemented with erythromycin (5 µg ml^−1^; Carl Roth, Karlsruhe, Germany), and TSA for LAC∆*sigB* and LAC* protease KO∆*sigB* mutants was supplemented with tetracycline (5 µg ml^−1^; Carl Roth). The characteristics of all isolates and the procedures followed to engineer the mutants are described in the supplementary material, tables S1, S4–S8 and figures S2 and S3.

### (b) Genome sequencing and molecular genetic typing

Comparative whole-genome sequencing analysis was performed as described previously [21]. For core genome (cg)MLST, we used the official scheme comprising 1861 target genes available in the Ridom SeqSphere+ software v.9.0.8 (Ridom, Münster, Germany) (https://www.cgmlst.org/ncs/schema/schema/141106/). A minimum spanning tree (MST) was then generated to visualize clusters and the genetic relatedness between the isolates, using a cluster threshold of 24 alleles. Locus-specific sequencing of the *sigB* operon (*rsbU*-*rsbV*-*rsbW*-*sigB*) was performed as previously described [23]. Genotyping was conducted by *spa* typing and multi-locus sequence typing (MLST) [48,49].

### (c) Growth media

Unless otherwise indicated, we used semi-skimmed milk (SSM) broth media, which consisted of a 1% w/v mixture of skimmed milk powder (LP0031, Thermo Fisher Scientific) in distilled water, equivalent to 10% fresh milk with less than or equal to 1.5% w/w fat content. SSM was sterilized by autoclaving at 121°C for 5 min. TSB was used for overnight cultures and growth control: it consisted of 30 g of powder (CM0129B, Thermo Fisher Scientific) dissolved in 1 l of distilled water and sterilized by autoclaving at 121°C for 15 min. Bacteria were grown in whole milk, specifically in 1% v/v commercially available fresh dairy milk (3.5% w/w fat content) diluted in distilled water. To study the influence of casein and lactose, the base minimal medium Roswell Park Memorial Institute Medium (RPMI-1640 without glucose; Sigma-Aldrich, Merck, Germany) was supplemented either with 1% casein sodium salt (Sigma-Aldrich) to obtain the C-medium, or with 1% D-lactose monohydrate (Sigma-Aldrich) to obtain the L-medium, or an equal mixture of 1% casein and lactose to obtain the C+L-medium.

### (d) Growth measurements

Bacteria were grown overnight (17–19 h) at 37°C in 3 ml TSB (preculture). Cell density was measured at 600 nm (OD_600_) in a cuvette using a photometer (Eppendorf, Hamburg, Germany). Bacterial cultures were diluted 1 : 5 in RPMI-1640 medium (Sigma-Aldrich) to minimize bacterial growth during handling and the transfer of the original TSB medium to the next growth medium. For the growth measurements taken in SSM, diluted bacterial cultures (approximately 10 µl) were used to inoculate SSM in a microplate, specifically 200 µl SSM per well; the final starting point for the experimental culture was OD_600_ = 0.05. In addition, diluted bacterial cultures were always transferred to fresh TSB as a parallel control. Plates were grown at 37°C without shaking for 72 h. Cell growth was automatically measured at OD_600_ every 30 min using Bioscreen-C (Oy Growth Curves Ab Ltd, Helsinki, Finland) after pre-shaking at medium intensity for 10 s. We used the same procedure to screen whole milk in 1% v/v, C-medium, L-medium and C+L-medium using a plate reader. In SSM, growth of the *S. aureus* strains TG13a, TG13o and TG13aR was also determined by counting colony-forming units (CFU). Viable cells were counted by gently disrupting clots and curd and spreading 10-fold serial dilutions in phosphate-buffered saline (PBS) (100 µl) on TSA plates in technical duplicates. After incubation at 37°C for 24 h, the CFU were determined using an automated colony counter (SphereFlash, IUL Instruments, Barcelona, Spain). The time of curd formation was determined visually as the time when curd first appeared at the bottom of wells. For some experiments, the pH was measured in the SSM media using a micro pH electrode (Thermo Fisher Scientific) every hour until the end of each experiment (up to 36 h). The calculation of the parameters—adaptation time τ and maximum (absolute) growth rate *ρ*_max_—is described in the supplementary material, method M1.

### (e) Carotenoid pigmentation and *asp23*-expression (real time-qPCR)

The carotenoid pigmentation of *S. aureus* was visualized and the absorbance of extracts of carotenoid pigmentation measured according to Walzl *et al.* [23]. For *asp23* expression, unless stated otherwise, bacterial strains were grown in TSB (120 r.p.m. and under aerobic conditions) and harvested at OD_600_ = 3, and *asp23* expression was determined following Marbach *et al.* [21] with specific primers for *asp23* [12] and normalized using three reference genes (*rpoD*, *rho* and *dnaN*). Mean relative expression and standard deviations were calculated from three independently grown samples and their technical duplicates (for experimental details, see supplementary material, method M2).

### (f) Proteolytic activity and ammonia, lactic acid and acetate assay

To determine the proteolytic activity in the culture media of TG13a (SigB-wt) and TG13o/TG13aR (SigB-def) in SSM, azocasein (A2765, Sigma-Aldrich), that is, casein linked to an azo-dye, was used as a substrate for proteolytic enzymes. The level of ammonia/ammonium ion, lactic acid and acetate were determined in the culture media of TG13a (SigB-wt) and TG13o/TG13aR (SigB-def) in SSM using the Ammonia Assay Kit MAK310-1KT (Sigma-Aldrich), the lactate assay kit K-LATE (Megazyme) and acetate assay kit K-ACETRM (Megazyme) according to the manufacturer's instructions. For the lactic acid and acetate assay, we used the Carrez clarification set K-CARREZ (Megazyme) to remove potentially interfering substances from the SSM sample. Technical duplicates of three independent experiments were analysed.

### (g) Statistical analysis

Analyses and graphical representations were conducted in either the GraphPad Prism 10 software (10.3.1) or the statistical programming language R [50]. Sample sizes of independent experiments are denoted as *n*, and data from independent experiments are always plotted. The values in the graphs for the growth curves are the mean growth at each time point plus/minus the standard deviation. When target variables were log- or logit-transformed to fulfil the assumptions of linear models this is explicitly mentioned. To compare mean growth values across more than two groups, we generally employed an analysis of variance (ANOVA) followed by correction for multiple comparisons (Tukey's honest significant difference or Dunnett test, as specified in each case). The competition experiment was analysed using a linear model (supplementary material, table S2). To account for sub-structuring of variability into clonal complexes and strains within clonal complexes, mixed models with strain and clonal complex (if appropriate) as additional regressors were performed. Significance was indicated by between one and four stars when the *p*-value was below 0.05, 0.01, 0.001 and 0.0001, respectively. Details of the statistical analyses are provided in the figure legends and the main text where they occur (see also supplementary material, method M3).

## Supplementary Material



## Data Availability

Data and code are available in Phaidra [51]. Supplementary material is available online [52].
