# Supplementary material for: A single transcriptional regulator is crucial for the adaptation of Staphylococcus aureus to diverse niches
Source: Proc Biol Sci. 2026 Oct 14;293(2080):20261930. doi: 10.1098/rspb.2026.1930 (PMC13678726; doi:10.1098/rspb.2026.1930)
Supplement: Supplementary file 1 [file rspb.2026.1930.zip › rspb.2026.1930/rspb20261930_si_001.pdf]

Supplementary Material for
Proceedings of the Royal Society B: Biological Sciences (Proc. R. Soc. B)
https://doi.org/10.1098/rspb.2026.1930

A single transcriptional regulator is crucial for the adaptation of *Staphylococcus aureus* to diverse niches

Claus Vogl,[1] Lynette C. Mikula,[2] Helene Marbach,[1] Katharina Mayer-Weihrauch,[1] Sebastian Herndler,[1] Leonie Breitschopf,[1] Amy C Pickering,[3] Mary-Elizabeth Jobson,[4,5] Adriana Cabal Rosel,[2] Nora Dinhopl,[1] Igor Loncaric,[1] Werner Rupitsch,[2,6,7] Lindsey N Shaw,[4,5] Orla Keane,[8] Ross Fitzgerald,[3] Monika Ehling-Schulz,[1] Simon Heilbronner,[9] Tom Grunert,[1]. Correspondence: tom.grunert@vetmeduni.ac.at

[1]Department for Biological Sciences and Pathobiology, University of Veterinary Medicine, Vienna, Austria
[2]Austrian Agency for Health and Food Safety (AGES), Vienna, Austria.
[3]The Roslin Institute, University of Edinburgh, Easter Bush, Midlothian, Edinburgh, United Kingdom
[4]Department of Molecular Biosciences, University of South Florida, Tampa, Florida, USA
[5]Center for Antimicrobial Resistance, University of South Florida, Florida, USA
[6]Institute of Hygiene and Medical Microbiology, Medical University Innsbruck, Austria.
[7]Faculty of Food Technology, Food Safety and Ecology, University of Donja Gorica, Podgorica, Montenegro
[8]Animal and Bioscience Department, Teagasc, Grange, Dunsany, Co. Meath, Ireland.
[9]Division of Microbiology, Ludwig-Maximilians-Universität München, Germany

This PDF file includes:

Supplementary Figures
Fig. S1. Comparison of TG13a (SigB-wt), and TG13o and TG13aR (both SigB-def)
Fig. S2. In-host evolution towards SigB-deficiency in bovine mastitis isolates of various clonal complexes
Fig. S3. Genetic relatedness of bovine-associated CC97 and CC151 isolates
Fig. S4. Screening of NTML regulatory mutant strains

Supplementary Tables
Table S1. Bovine-associated isolates from persistent IMI and engineered mutants
Table S2. Logistic regression analysis of the competition experiment of TG13o vs TG13a
Table S3. Average difference between TG13a, TG13o, and TG13aR of lactose consumption, production of metabolites, pH, and proteolytic activity after 8 h and 24 h of growth in SSM (*p*-values of t-tests shown)
Table S4. Bovine isolates for comparison of CC97 and CC151
Table S5. Human-associated isolates (CC8) and engineered mutants
Table S6. *S. aureus* isolates for comparison of human- and bovine-associated CC
Table S7. *S. aureus* Nebraska Transposon Mutant Library (NTML)
Table S8. The oligonucleotides employed for genetic manipulation in this research

Supplementary Methods
Supplementary Method M1. Calculation of the parameters adaptation time τ and maximum (absolute) growth rate ρ_{max}
Supplementary Method M2. *Asp23* expression (RT-qPCR)
Supplementary Method M3. Handling Poorly Growing Strains in Statistical Analyses

Fig. S1.

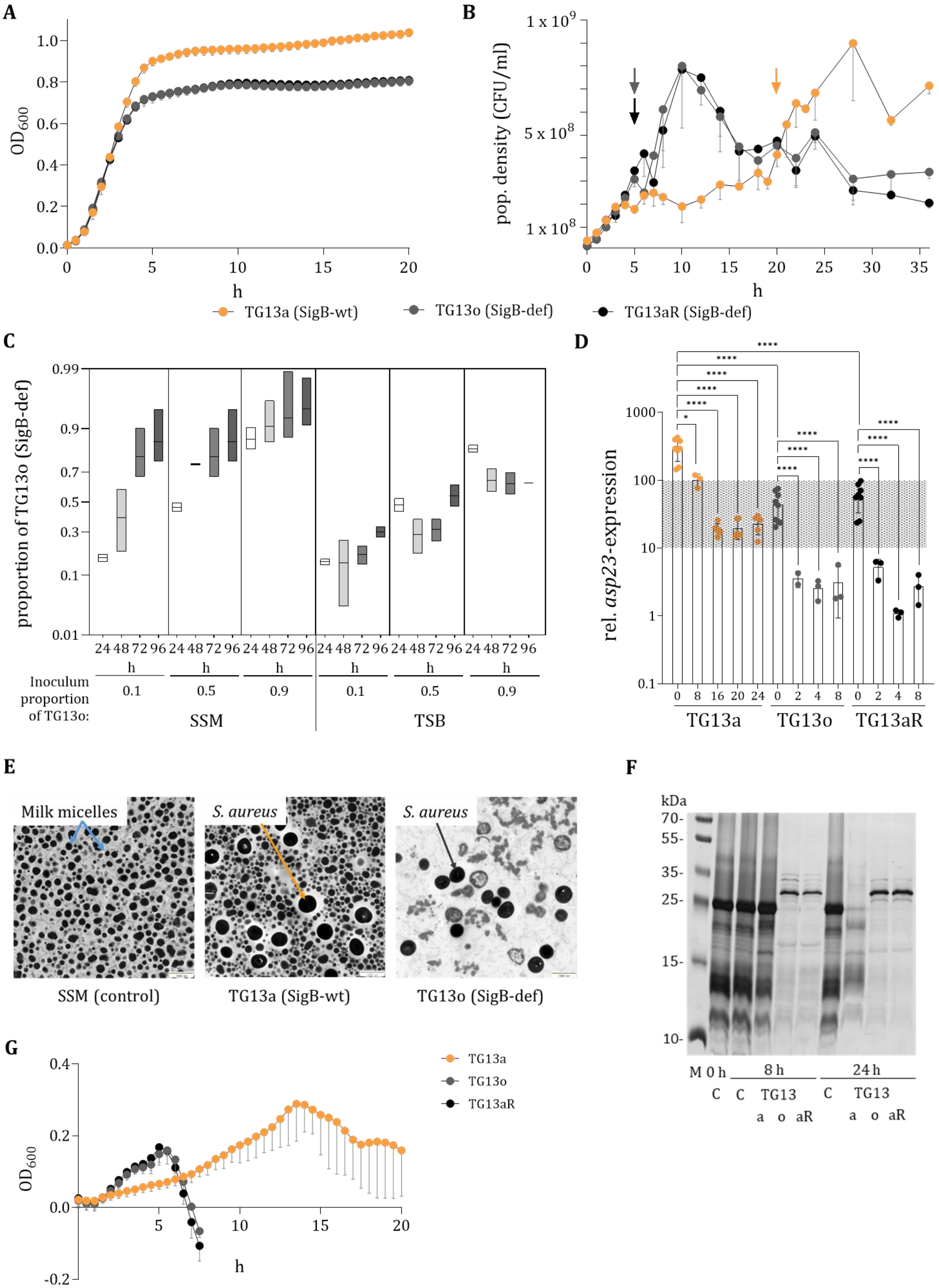

https://doi.org/10.1098/rspb.2026.1930

Fig. S1. Comparison of TG13a (SigB-wt), and TG13o and TG13aR (both SigB-def). (A) *S. aureus* strains pre-cultivated in TSB and then transferred to fresh TSB grow without an adaptation phase. **(B)** Population density in CFU/ml after transfer to SSM. The arrows mark the time of curd formation. **(C)** Competition experiment TG13o vs TG13a. Boxplots of the proportion of the derived SigB-deficient strain TG13o after 24, 48, 72 and 86 h in SSM and TSB, depending on the proportion of TG13o (vs TG13a) in the initial inoculum. We test if the *in vivo* evolved SigB-def strain (TG13o) has a growth advantage over the initially dominant SigB-wt strain (TG13a) after transfer from TSB to SSM by co-cultivating the strains from different starting proportions of the inoculum (0.1, 0.5, and 0.9 of TG13o). Concurrently, a control experiment involving transfer of the same inocula to fresh TSB is performed. After 24, 48, 72 and 96 h, yellow and white CFUs are counted to determine the proportion of TG13o. In total, four independent iterations of this experiment are conducted, two for early (24 and 48 h) and two for late time points (72 and 96 h). (**D**) Relative *asp23* mRNA expression (RT-qPCR; mean ± SD) in the interval (h) after transfer from TSB to SSM. Differences in pairwise means were tested with Tukey's HSD. The overlapping range of SigB activity in SigB-wt and SigB-def strains is underlaid. **(E)** Transmission Electron Microscopy (TEM) images of SSM without bacteria (media control), and after growth for 24 h in SSM. For TEM, bacterial and control samples were fixed, dehydrated, and embedded in resin before being cut into ultrathin sections and stained. The prepared sections were then examined using a Zeiss TEM 900 microscope operated at 50 kV. **(F)** Sodium dodecyl sulfate(SDS)-polyacrylamide gel electrophoresis and silver staining were performed to visualise the SSM degradation pattern induced by TG13a (SigB-wt) and TG13o/TG13aR (SigB-def) using an isocratic 15%T, 18 cm x 16 cm separation gel. Media controls without bacteria, labelled C, are included at time points t = 0, t = 8 and t =24. Sample load was normalised to equal protein concentration. **(G)** The growth curves (OD_{600}) of TG13a (SigB-wt), TG13o, and TG13aR (both SigB-def) after transfer from TSB to fresh milk with 3.5% fat content (OD_{600}; mean – SD).
M, Marker proteins. TG13a, SigB-wt, TG13o and TG13aR, both SigB-def.

https://doi.org/10.1098/rspb.2026.1930

Fig. S2.

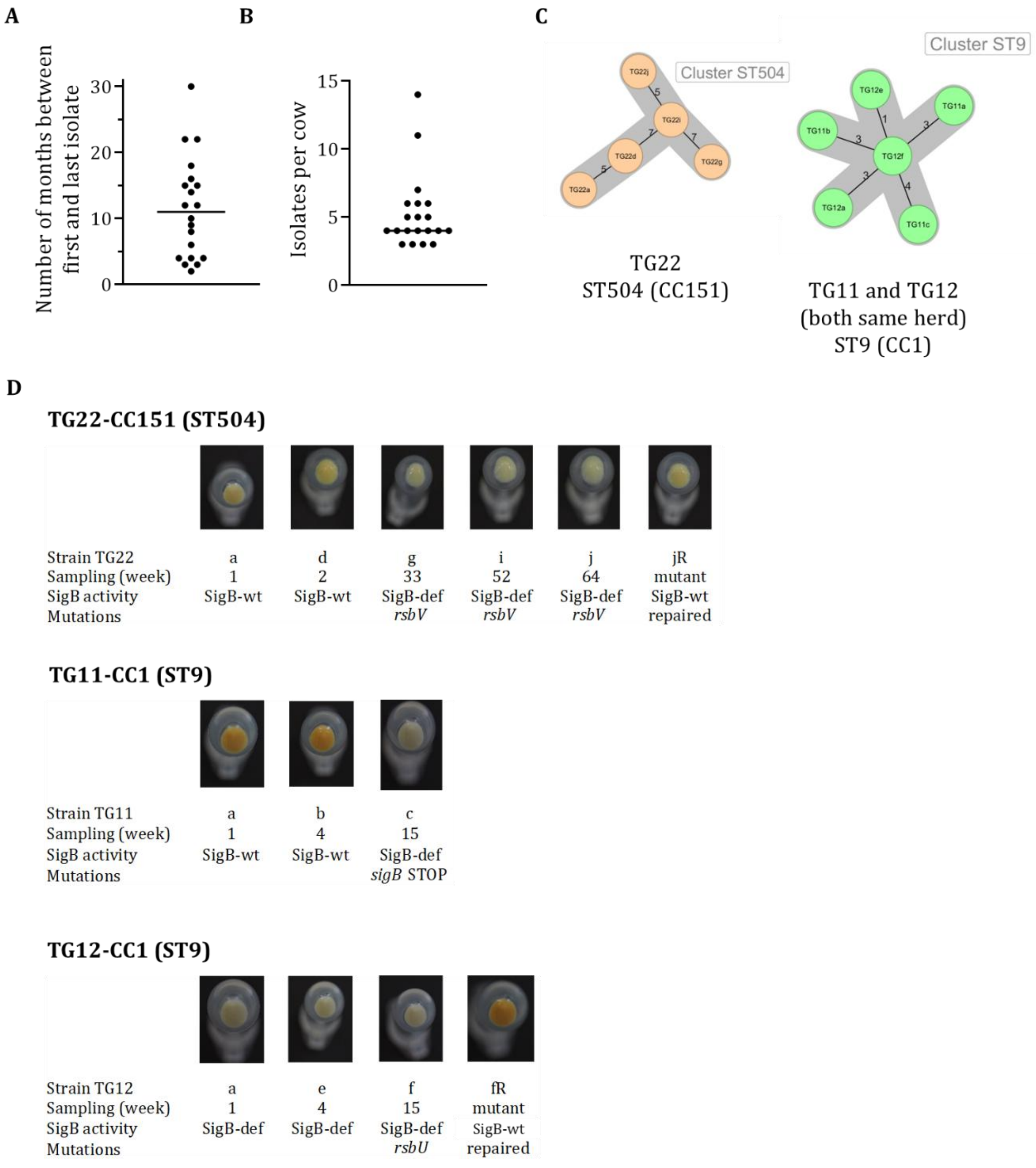

In-host evolution towards SigB-deficiency in bovine mastitis isolates of various clonal complexes. (A) Intervals between the first and the last isolate collected from individual dairy cows (n = 20), and **(B)** number of isolates collected from each cow (from which a minimum of three isolates was obtained) by retrospective screening for SigB-def identifiable by visible loss of colony pigmentation (i.e., for a white or grey instead of a yellow colony colour) from consecutively sampled isolates of *S. aureus* from bovine hosts with persistent IMI (n = 20). **(C)** Putative SigB-

https://doi.org/10.1098/rspb.2026.1930

def isolates in milk samples from three cows from two herds. Minimum spanning tree using core genome multilocus sequence typing (cgMLST) data for TG22 and TG11/TG12 (same herd) suggests clonality, although the isolates were recovered from different cows, suggesting transmission from one cow to the other. We used a cluster threshold of ≤24 allelic differences in the core genome to determine clonality. For TG22, the first and last isolates were collected 15 months apart. Both of these, as well as the three intermediate isolates collected from the same cow, have a cgMLST cluster of less than 24 allelic differences, indicating clonality. Isolates were assigned to the clonal complexes CC97, CC151, and CC1. **(D)** Isolate sampling and SigB activity: In both reconstructed strains (TG22jR and TG12fR) carotenoid pigmentation is restored, confirming that the mutations are solely responsible for the inactivation of the *sigB* operon.

https://doi.org/10.1098/rspb.2026.1930

Fig. S3.

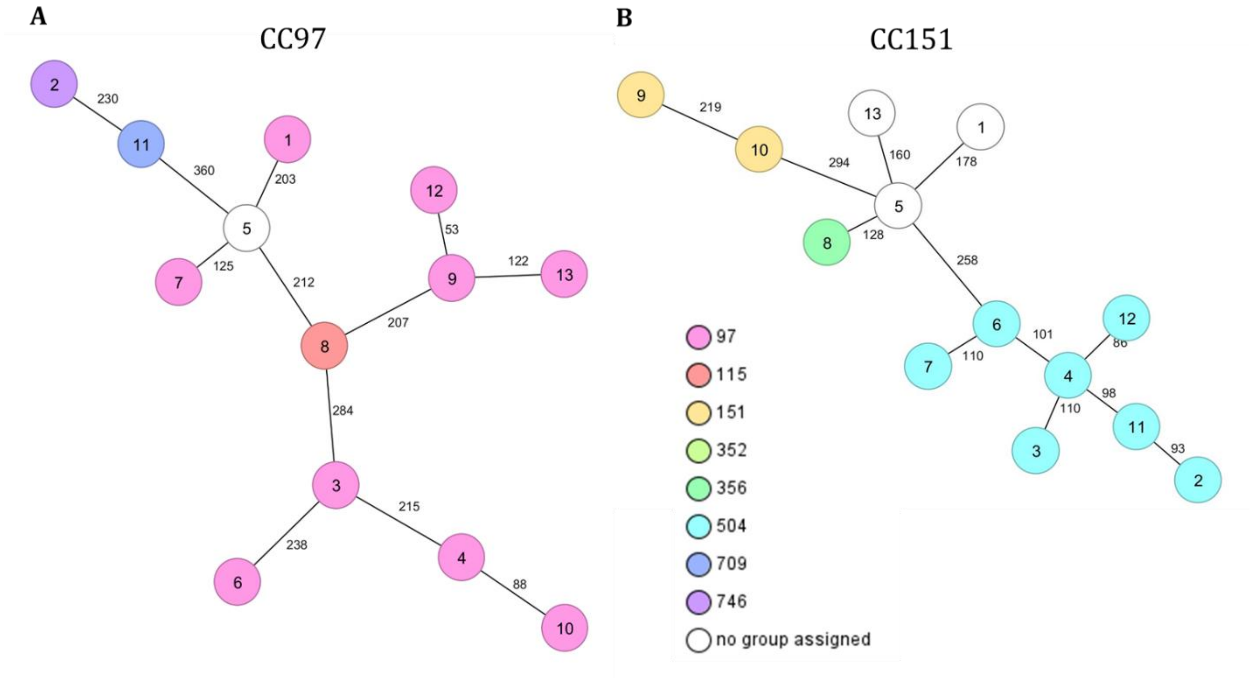

Genetic relatedness of bovine-associated CC97 and CC151 isolates. Minimum spanning tree using core genome multilocus sequence typing (cgMLST) data for strains of **(A)** CC97 (n = 13) and **(B)** CC151 (n = 13) isolated from the milk of cows from different geographic regions (see the list of strains and their characteristics in Supplementary Table 4). Graphical representation of their relationships obtained by a cgMLST analysis. All isolates show unique cgMLST patterns, exceeding the threshold of ≤24 allelic differences in the core genome used to determine clonality. The colour of the nodes indicates the ST type.

https://doi.org/10.1098/rspb.2026.1930

Fig. S4.

A

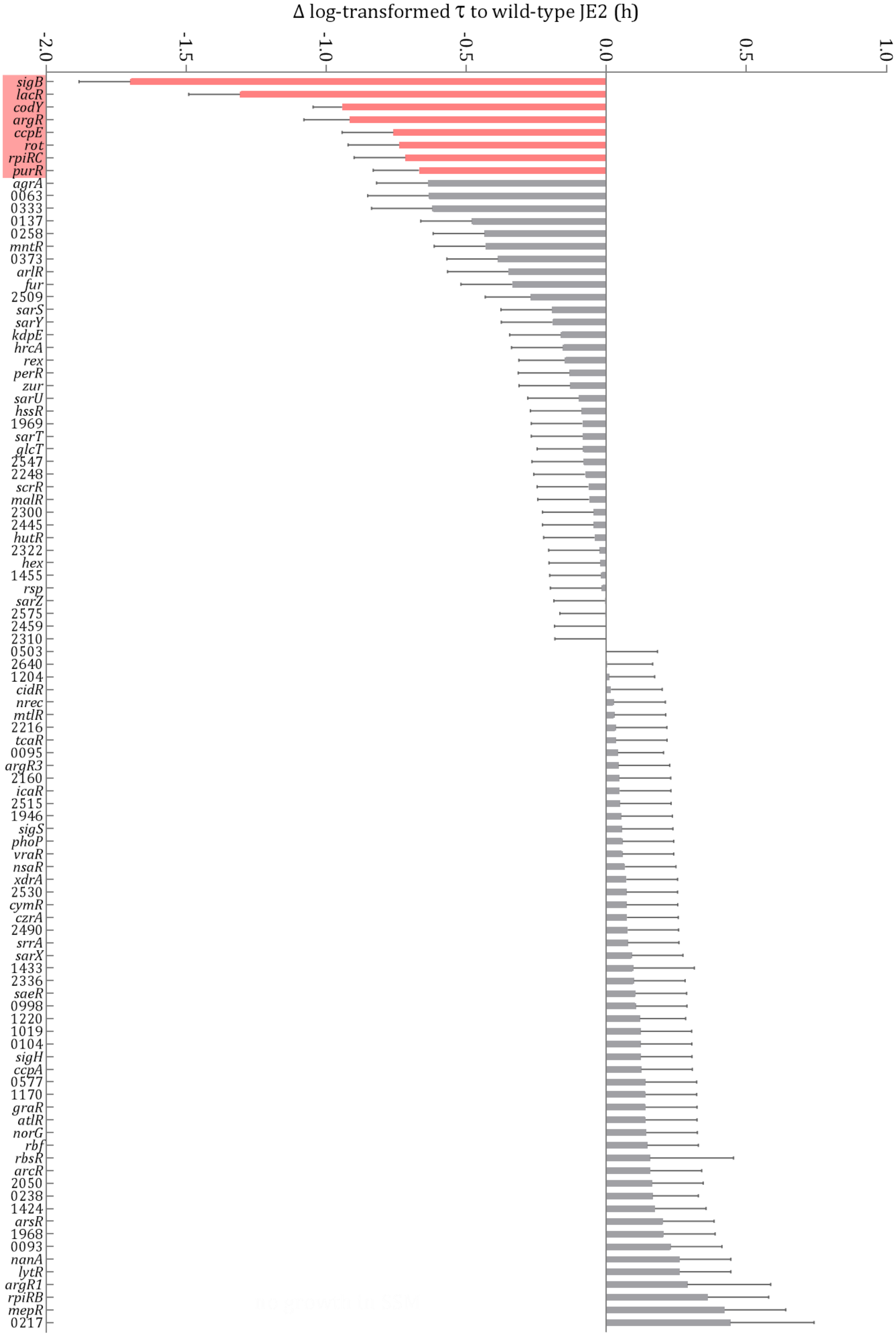

https://doi.org/10.1098/rspb.2026.1930

Fig. S4

B

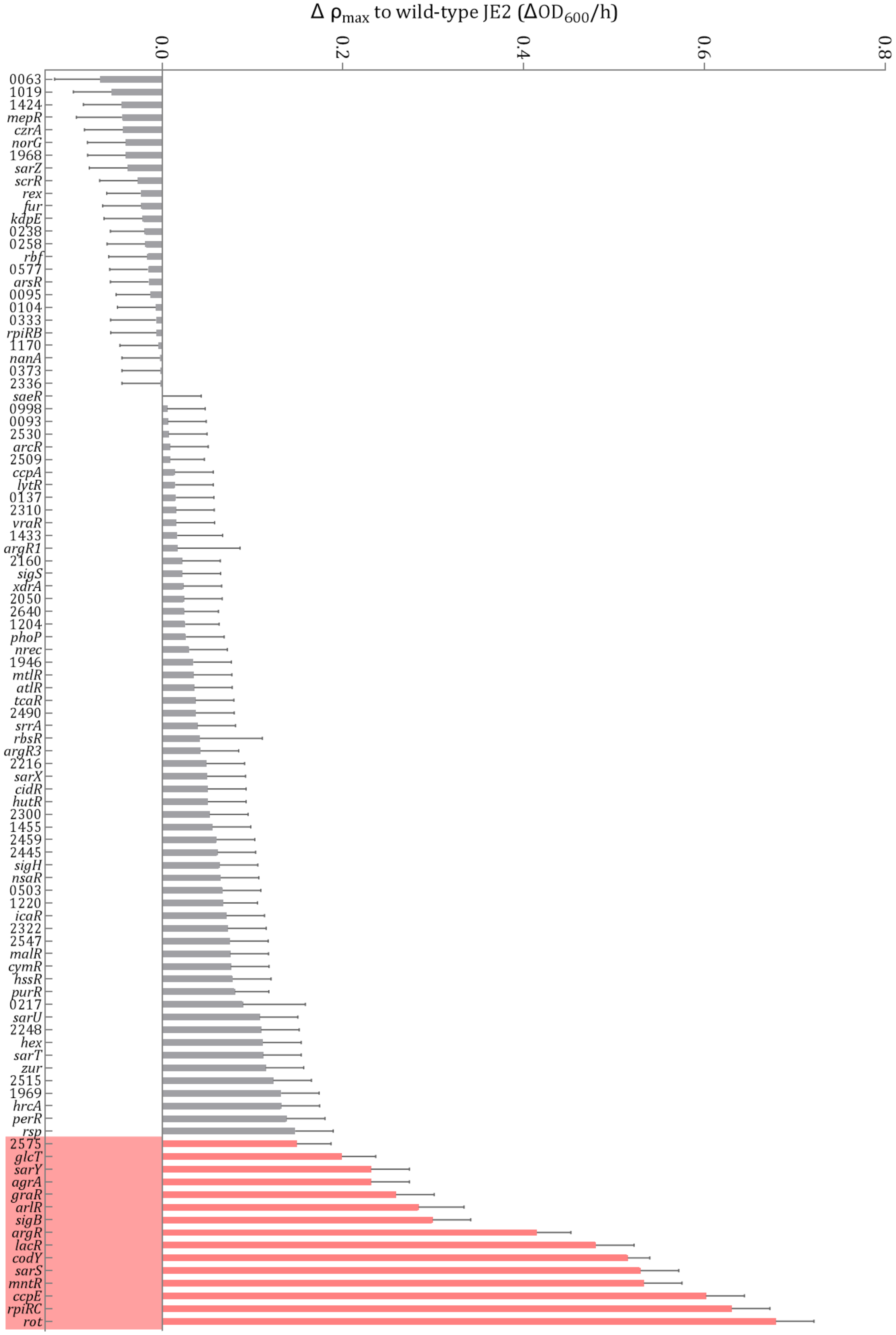

https://doi.org/10.1098/rspb.2026.1930

Fig. S4.

C

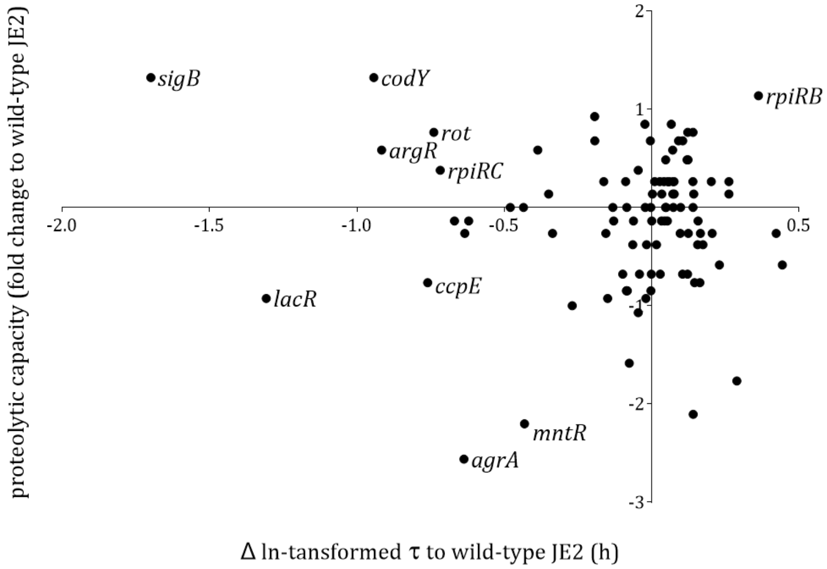

D

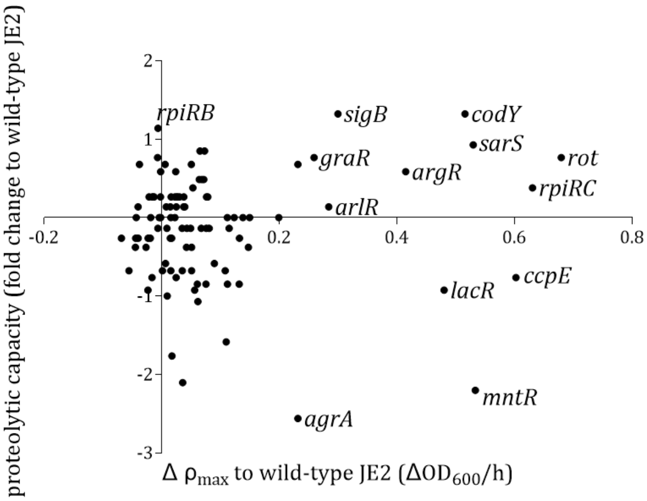

Fig. S4. Screening of NTML regulatory mutant strains. Means and standard errors of the differences between each NTML mutant strain and the wild-type reference strain with respect to **(A)** adaptation time (τ) and **(B)** maximal growth rate (ρ_{max}). The pink colour indicates significant differences as determined by Dunnett's test with the wild-type as a reference. The four mutants *gltC*, *treR*, *sarA,* and NE40 (SAUSA300_1174) were excluded from the analysis because they did not grow in SSM within 72 h. For each NTML regulatory mutant strain, the difference in proteolytic capacity from the reference JE2 strain (on the x-axis) is plotted **(C)** vs the difference in the ln-transformed adaptation time (τ), and **(D)** the maximal growth rate (ρ_{max}) (y-axis) from the reference JE2 strain. The pairwise Pearson correlations are not significant and therefore not shown.

https://doi.org/10.1098/rspb.2026.1930

Table S1.

Bovine-associated isolates from persistent IMI and engineered mutants

Strain	Description	SigB activity[1]	Sampling week	CC	ST	*spa*-type	*cap*-*gene*	*agr*-type	Reference
TG13a	First isolate (IN)	+	1						Marbach *et al.* 2019
TG13aΔ*sspA*	*sspA* mutant of TG13a	+							this study
TG13aΔ*aur*	*aur* mutant of TG13a	+							this study
TG13o	21th and last isolate (HA), incl. mutation in *rsbU* (G368A)	-	14						Marbach *et al.* 2019
TG13oΔ*sspA*	*sspA* mutant of TG13o	-		97	352	t044	5	*I*	this study
TG13oΔ*aur*	*aur* mutant of TG13o	-							this study
TG13aR	TG13a::*rsbU* (G368A) mutant	-							Walzl *et al.* 2023
TG13aRΔ*sspA*	*sspA* mutant of TG13aR	-							this study
TG13aRΔ*aur*	*aur* mutant of TG13aR	-							this study
**TG11-CC1**									
TG11a	First isolate	+	1						this study
TG11b	Second isolate	+	4	1	9	t3446	5	*II*	this study
TG11c	Third and last isolate, incl. stop codon in *sigB*	-	15						this study
**TG12-CC1**									
TG12a	First isolate, incl. SNP G431T in *rsbU*	-	1						this study
TG12e	Second isolate, incl. SNP G431T in *rsbU*	-	4	1	9	t3446	5	*II*	this study
TG12f	Third and last isolate, incl. SNP G431T in *rsbU*	-	15						Walzl *et al.* 2023
TG12fR	TG12f::*rsbU* repaired mutant	+							Walzl *et al.* 2023
**TG22-CC151**									
TG22a	First isolate	+	1						Grunert *et al.* 2018
TG22d	Second isolate	+	2						Grunert *et al.* 2018
TG22g	Third isolate, incl. 62bp trunc. in *rsbV*	-	33	151	504	t529	8	*II*	Grunert *et al.* 2018
TG22i	Fourth isolate, incl. 62bp trunc. in *rsbV*	-	52						Grunert *et al.* 2018
TG22j	Fifth and last isolate, incl. 62bp trunc. in *rsbV*	-	64						Grunert *et al.* 2018
TG22jR	TG22j::*rsbV* repaired mutant	+							this study

1) +, SigB-wt; -, SigB-deficient

https://doi.org/10.1098/rspb.2026.1930

Table S1

Bovine-associated isolates from persistent IMI and genetically engineered mutants

The isolates TG13a (SigB-wt) and TG13o (SigB-def) were described in our previous study (Marbach et al., 2019). The single nucleotide change *rsbU*(G368A) observed in TG13o was introduced into TG13a, and the resulting strain designated as TG13aR (Walzl et al., 2023). In addition, we engineered isogenic knockout mutants of the proteases Aur (Δ*aur*) and SspA (Δ*sspA*) based on the SigB-wt (TG13a) and SigB-def strains (TG13o/ TG13aR). The thermosensitive plasmid pIMAY-Z was utilised to generate *aur* and *sspA* deletion mutants using previously described protocols (Monk et al., 2012). Briefly, oligonucleotide primers were used to amplify ~500 bp AB and CD flanking regions of each gene of interest (Supplementary Table S8) using Q5 polymerase (NEB). Gibson assembly (NEB) generated pIMAY-Z constructs that were transformed firstly into *E. coli* DC10B (Monk et al., 2012) and then, after Sanger sequencing confirmation (Eurofins), into the *S. aureus* strain of interest. After integration at 37°C and plasmid excision at 30°, OUT PCR was used to confirm that the clones contained gene deletions.

The isolates TG11-CC1 (n = 3), TG12-CC1 (n = 3) and TG22-CC151 (n = 5) were obtained by retrospectively screening 105 staphylococcal isolates from the milk of 20 cows with persistent IMI for visible loss of colony pigmentation (i.e., for a white or grey instead of a yellow colour). Note that we consider IMI persistent when clonal isolates can be collected from at least three consecutive milk samples from the same dairy cow, with a minimum of one month between the first and the last of these three samples. This screen yielded putative SigB-def isolates in milk samples from three cows from two herds. The isolates TG22-CC151 were from one udder quarter of a single cow (cow 11 in study Grunert, et al. 2018): Isolates from samples taken within the first two weeks (TG22a and d) are SigB-wt, while those from samples taken in weeks 33 (TG22g), 52 (TG22i), and 64 (TG22j) are SigB-def. The isolates TG11-CC1 (n = 3) and TG12-CC1 (n = 3) came from cows of the same dairy herd in Austria, and were collected from milk samples taken during routine microbial mastitis diagnostic screening over 15 weeks. In fact, TG11-CC1 and TG12-CC1 isolates were collected at weeks 1, 4 and 15 from two different cows. While TG11a and TG11b are SigB-wt, a stop codon in the *sigB* gene of TG11c causes SigB-deficiency. All three TG12 isolates (a, e, and f) are SigB-def due to the SNP G431T in *rsbU*. Genome sequencing of these isolates reveals clonality within isolates from the same cow, indicating persistent infection with the same strain. Although the isolates TG11 and TG12 were recovered from different cows, genome sequencing indicates clonality, suggesting transmission from one cow to the other. We also engineered the following isogenic mutant strains: In strain TG12fR we reversed the point mutation *rsbU*:431G→T (Walzl et al., 2023). To restore the 62 bp truncation in *rsbV* TG22j, the full-length allele was amplified (Supplementary Table S8) from the precursor isolate TG22a and cloned into pIMAY. Allelic exchange was performed as described above (Monk et al., 2012). The resulting strain was named TG22jR.

https://doi.org/10.1098/rspb.2026.1930

Table S2.

Logistic regression analysis of the competition experiment of TG13o vs TG13a: Inncolum with different proportions of TG13o (0.1, 0.5, 0.9) was grown in both TSB and SSM, and the growth of each strain was evaluated at different time points (24h, 48h, 72h, 96h). The dependent variable in the regression analysis is the log-odds of the observed strain proportions. This is regressed onto the independent variables encoding the medium (TSB corresponds to 0; SSM to 1), the evaluation times, and the log-odds of the initial strain proportions (note this precludes inclusion of an intercept). Including a random effect term for different replicates does not improve model fit (not shown).

```
Call:
lm(formula = logit(proportion) ~ 0 + times * medium * logit(init pr),
    data = df_biol)
Residuals:
    Min      1Q  Median      3Q     Max
-1.5657 -0.4528  0.1640  0.4504  1.6048
Coefficients:
                                 Estimate Std. Error t value Pr(>|t|)
times                            0.001748   0.005398   0.324 0.747714
medium_TSB                      -0.508199   0.354815  -1.432 0.159830
medium_SSM                      -0.565521   0.354815  -1.594 0.118843
logit(init pr)                   0.915966   0.197776   4.631 3.8e-05  ***
times:medium_SSM                 0.028384   0.007634   3.718 0.000615 ***
times:logit(init pr)            -0.006171   0.003009  -2.051 0.046875 *
Medium_SSM:logit(init pr)        0.012214   0.279697   0.044 0.965385
times:medium_SSM:logit(init pr)  0.001241   0.004255   0.292 0.772111
---
Signif. codes:  0 '***' 0.001 '**' 0.01 '*' 0.05 '.' 0.1 ' ' 1
Residual standard error: 0.7096 on 40 degrees of freedom
Multiple R-squared:  0.8499,    Adjusted R-squared:  0.8199
F-statistic: 28.31 on 8 and 40 DF,  p-value: 3.759e-14
```

https://doi.org/10.1098/rspb.2026.1930

Table S3.

Average difference between TG13a, TG13o, and TG13aR of lactose consumption, production of metabolites, *pH,* and proteolytic activity after 8 h and 24 h of growth in SSM (*p-* values of t-tests shown)

	8_p_overall	8_p_13o-13a	8_p_13rA-13a	8_p_13rA-13o	24_p_overall	24_p_13o-13a	24_p_13rA-13a	24_p_13rA-13o
**pH**	2.843171e-03	4.499992e-03	5.008966e-03	0.993082793	9.619389e-04	0.0018166542	0.0015131405	0.9733890
**acetate**	2.002997e-04	4.349719e-04	2.957351e-04	0.831265171	5.130239e-04	0.0009346894	0.0008547612	0.9922753
**lactate**	2.521679e-05	4.469903e-05	4.614951e-05	0.997530486	7.809455e-05	0.0001280476	0.0001530213	0.9474261
**proteolytic activity**	1.334007e-04	2.334014e-04	2.408150e-04	0.998577416	4.161395e-03	0.0056157065	0.0087043103	0.7803790
**ammonia**	3.153807e-06	2.723864e-06	1.952173e-05	0.004176723	1.009559e-04	0.0003202220	0.0001193526	0.2958623

https://doi.org/10.1098/rspb.2026.1930

Table S4.

Bovine isolates for comparison of CC97 and CC151

CC	ID	ST	*spa*- type	*cap* - gene	*agr*- type	Strain	Origin[1]	Reference
	1	ST97	t267			A1	ARG	Grunert *et al.* 2013
	2	ST746	t267			A4	ARG	Grunert *et al.* 2013
	3	ST97	t2112			39FR	RWA	Antok *et al.* 2019
	4	ST97	t1236			87RL	RWA	Antok *et al.* 2019
	5	ST97	t521			MB328	ARG	Tuchscherr *et al.* 2007
	6	ST97	t10103			101RR	RWA	Antok *et al.* 2019
CC97	7	ST97	t521	5	*I*	A10	ARG	Grunert *et al.* 2013
	8	ST115	t044			N305	CAN	Prasad *et al.* 1968
	9	ST97	t044			S413-1	AUT	Kümmel *et al.* 2016
	10	ST97	t9432			37RR	RWA	Antok *et al.* 2019
	11	ST709	t267			MB325	ARG	Tuchscherr *et al.* 2007
	12	ST97	t044			S387-2	AUT	Kümmel *et al.* 2016
	13	ST97	t267			127	AUT	Schabauer *et al.* 2018
	1	ST705	t529			A20	ARG	Grunert *et al.* 2013
	2	ST504	t529			20	AUT	Schabauer *et al.* 2018
	3	ST504	t529			14c	AUT	this study
	4	ST504	t529			38	AUT	Schabauer *et al.* 2018
	5	ST705	t529			A18	ARG	Grunert *et al.* 2013
	6	ST504	t529			S392-1	AUT	Kümmel *et al.* 2016
CC151	7	ST504	t529	8	*II*	S429	AUT	Kümmel *et al.* 2016
	8	ST356	t529			A14	ARG	Grunert *et al.* 2013
	9	ST151	t529			115	AUT	Schabauer *et al.* 2018
	10	ST151	t529			RF122	GBR	Fitzgerald *et al.* 2000
	11	ST504	t529			LMM-22	AUT	this study
	12	ST504	t529			W18c	AUT	Grunert *et al.* 2018
	13	ST705	t458			A15	ARG	Grunert *et al.* 2013

1) IRL, Irland; ARG, Argentina; RWA, Rwanda; CAN, Canada; AUT; Austria; GBR, United Kingdom.

CC151	MOK124-CC151	ST151	nd	8	*II*	MOK124	IRL	Murphy *et al.* 2019
CC97	MOK023-CC97	ST3170	nd	5	*I*	MOK023	IRL	Murphy *et al.* 2019

https://doi.org/10.1098/rspb.2026.1930

Table S4

Bovine isolates for the comparison of CC97 and CC151

We selected the strain pair MOK023-CC97 and MOK124-CC151 because comprehensive *in vivo* and *in vitro* data are available (Murphy, Niedziela, Leonard, & Keane, 2019; Niedziela, Cormican, Foucras, Leonard, & Keane, 2021; Niedziela, Murphy, Grant, Keane, & Leonard, 2020). MOK023-CC97 (ST3170) and MOK124-CC151 (ST151) strains were originally isolated from cases of clinical mastitis (Keane, Budd, Flynn, & McCoy, 2013). The CC97 and CC151 isolates (n = 13, each) used for comparative analyses in this article originate from our collection of well-characterised strains from bovine mastitis milk samples (Antók et al., 2019; Fitzgerald, Hartigan, Meaney, & Smyth, 2000; Tom Grunert et al., 2018; T. Grunert et al., 2013; Kummel et al., 2016; Prasad & Newbould, 1968; Schabauer et al., 2018; L. P. Tuchscherr et al., 2007). Strains were selected to represent diverse geographic origins (Austria, n = 11; Argentina, n = 9; Rwanda, n = 4; others, n = 2), sequence types (STs), and *spa*-types. Core genome multilocus sequence typing (cgMLST) and subsequent analyses are described in the subsection: Genome sequencing and molecular genetic typing. An overview of cgMLST analyses of all strains is provided in Supplementary Fig. S3.

https://doi.org/10.1098/rspb.2026.1930

Table S5.

Human-associated isolates (CC8) and engineered mutants

Strain	Description	SigB activity[1]	CC	ST	*spa*-type	*cap*-gene	*agr*-type	Reference
Nm	Newman - human infection	+	8	254	t008	5	nd	Duthi *et al.* 1952
NmR	Newman::*rsbU* (G368A) mutant	-						Walzl *et al.* 2023
8325-4	11 bp deletion in *rsbU* gene	-						Novick *et al.* 1967
SH1000	*rsbU* repaired mutant	+	8	8	t211	5	nd	Horsburgh *et al.* 2002
SH1000Δ*sigB*	*sigB* deletion mutant of SH1000	-						Tuchscherr *et al.* 2015
LAC	USA300 LAC (AH1263)	+						Boles *et al.* 2010
LACΔ*sigB*	USA300 LAC *sigB*::tet (LNS2602)	-	8	8	nd	5	nd	this study
ESPNΔ*sigB*	USA300 LAC Δ*aur* Δ*sspAB* Δ*scpA* spl::erm *sigB*::tet (LNS4021)	-						this study

1) +: SigB-wt; -: SigB-deficient

https://doi.org/10.1098/rspb.2026.1930

Table S5

Human-associated isolates and genetically engineered mutants

Strain 8325-4 is a widely used model strain originally isolated from a sepsis patient in 1960 (strain RN1 or NCTC8325); all three prophages were cured by UV radiation (Novick, 1967). Strain 8325-4 is SigB-def because of an 11-bp deletion in *rsbU*, which was repaired in strain SH1000 (SigB-wt) (Horsburgh et al., 2002). The isogenic mutant SH1000Δ*sigB* (SigB-def) was derived from SH1000 (L. Tuchscherr et al., 2015). Strain Newman (Nm, NCTC8178) is SigB-wt and was isolated from a patient with osteomyelitis (Duthie & Lorenz, 1952). We introduced the same single nucleotide change in *rsbU*(G368A) that we studied in TG13o to the Newman strain to make the SigB-def mutant strain NmR (Walzl et al., 2023). The SigB-wt strain LAC (CA-MRSA USA300) was originally isolated from a human skin and soft tissue infection (Voyich et al., 2005), and we engineered the isogenic mutant LACΔ*sigB* (SigB-def) from it. In addition, we combined a derivative of the LAC strain that lacks all 10 major secreted proteases (LAC*protease KO) with a SigB-def mutant, LAC* protease KOΔ*sigB*. The protease null strain (Δ*aur*Δ*sspAB*Δ*scpAspl*::erm) was originally generated by (Wörmann, Reichmann, Malone, Horswill, & Gründling, 2011) and the originating *sigB*::tet mutation was generated by Chan et al., 1998. Strains were constructed using Φ11 phage lysates containing *sigB*::tet and transduced into either the USA300 LAC background (Boles, Thoendel, Roth, & Horswill, 2010), or the USA300 LAC protease null background. Transduced strains were selected on TSA containing 5 µg/mL tetracycline and confirmed via PCR using the primers OL7735 and OL7736.

https://doi.org/10.1098/rspb.2026.1930

Table S6.

***S. aureus* isolates for comparison of human- and bovine-associated CC**

Host	CC	ST	*spa*-type	*cap*-gene	*agr*-type	Strain	Reference
HUMAN	CC8	ST250	t008	5	*I*	Newman	Duthi *et al.* 1952
	CC8	ST254	t008	5	*I*	COL	Dyke *et al.* 1966
	CC8	ST8	t648	5	*I*	52	Johler *et al.* 2016
	CC8	ST72	t148	5	*I*	29	Johler *et al.* 2016
	CC30	nd	t018	5	*III*	16	Johler *et al.* 2016
	CC30	ST30	t021	5	*III*	43	Johler *et al.* 2016
	CC30	ST36	t018	5	*III*	37	Johler *et al.* 2016
	CC5	ST4255	t8456	5	*II*	39	Johler *et al.* 2016
	CC5	ST5	t857	5	*II*	57	Johler *et al.* 2016
	CC5	ST5	t179	5	*II*	38	Johler *et al.* 2016
	CC45	ST508	t630	8	*I*	19	Johler *et al.* 2016
	CC45	ST45	t950	8	*I*	27	Johler *et al.* 2016
	CC45	ST45	t445	8	*I*	68	Johler *et al.* 2016
BOVINE	CC151	ST504	t529	8	*II*	S392-1	Kümmel *et al.* 2016
	CC151	ST151	t529	8	*II*	RF122	Fitzgerald *et al.* 2000
	CC151	ST504	t529	8	*II*	14c	this study
	CC97	ST115	t044	5	*I*	N305	Prasad *et al.* 1968
	CC97	ST709	t267	5	*I*	MB325	Tuchscherr *et al.* 2007
	CC97	ST97	t044	5	*I*	S387-2	Kümmel *et al.* 2016
	CC8	ST8	t2953	5	*I*	S276-2	Kümmel *et al.* 2016
	CC8	ST8	t2953	5	*I*	LMM-216	this study
	CC8	ST8	t2953	5	*I*	108	Schabauer *et al.* 2018
	CC8	ST8	t2953	5	*I*	171	Schabauer *et al.* 2018

https://doi.org/10.1098/rspb.2026.1930

Table S6

***S. aureus* isolates for comparison of human- and bovine-associated CCs**

A set of 13 human- and 10 bovine-associated isolates was used. The subset of human isolates includes major MRSA and MSSA epidemic clones, specifically: Four isolates of CC8, and three isolates of CC30, CC5, and CC45 each. These represent CCs commonly isolated from asymptomatic and infected humans (Duthie & Lorenz, 1952; Dyke, Jevons, & Parker, 1966; Johler, Stephan, Althaus, Ehling-Schulz, & Grunert, 2016). The subset of bovine-associated isolates comprises three strains each of CC97 and CC151, and four isolates of bovine CC8 (Fitzgerald et al., 2000; Kummel et al., 2016; Prasad & Newbould, 1968; Schabauer et al., 2018; L. P. Tuchscherr et al., 2007). Within each CC, strains were selected to represent diverse sequence types (ST, MLST) and *spa*-types.

https://doi.org/10.1098/rspb.2026.1930

Table S7.

***S. aureus* Nebraska Transposon Mutant Library (NTML)**
NTML mutants are based on human-derived, wild-type JE2, a plasmid-cured variant of USA300 LAC (CC8)

#	Gene ID SAUSA300_	gene name	proteolytic capacity (log_2 fold change compared to WT) after Giemza *et al.* 2019
1	0063	0063	-0,2630
2	0066	argR1	-1,7655
3	0093	0093	-0,5850
4	0095	0095	0,2630
5	0104	0104	0,7655
6	0110	norG	0,1375
7	0114	sarS	0,9260
8	0137	0137	0,0000
9	0195	rpiRB	1,1375
10	0217	0217	-0,5850
11	0238	0238	-0,2630
12	0255	lytR	0,1375
13	0258	0258	0,0000
14	0265	rbsR	-0,3785
15	0315	nanA	0,2630
16	0333	0333	-0,1375
17	0334	mepR	-0,2630
18	0373	0373	0,5850
19	0473	purR	-0,1375
20	0503	0503	-0,1375
21	0519	sigH	0,4854
22	0577	0577	0,2630
23	0621	mntR	-2,2016
24	0645	graR	0,7655
25	0653	rbf	-0,7655
26	0654	sarX	0,6781
27	0658	ccpE	-0,7655
28	0691	saeR	-0,6781
29	0954	atlR	-2,1043
30	0998	0998	0,6781
31	1019	1019	-0,6781
32	1148	codY	1,3219
33	1170	1170	0,0000
34	1204	1204	0,2630
35	1220	1220	0,4854
36	1253	1253	0,0000
37	1308	arlR	0,1375
38	1409	vraR	0,2630
39	1424	1424	-0,3785
40	1433	1433	-0,2630
41	1442	srrA	0,1375
42	1448	fur	-0,2630
43	1455	1455	-0,9260
44	1457	malR	-0,1375
45	1469	argR	0,5850
46	1514	zur	-0,1375
47	1542	hrcA	-0,2630
48	1583	cymR	0,2630
49	1639	phoP	0,2630
50	1682	ccpA	-0,2630
51	1708	rot	0,7655
52	1717	arsR	0,2630
53	1722	sigS	0,2630
54	1797	xdrA	0,5850
55	1842	perR	0,0000
56	1946	1946	-0,1375
57	1968	1968	-0,2630
58	1969	1969	-0,8480
59	1992	agrA	-2,5607
60	1995	scrR	-0,3785
61	1999	rex	-0,9260
62	2022	sigB	1,3219
63	2036	kdpE	0,2630
64	2050	2050	-0,7655
65	2098	czrA	0,0000
66	2106	mtlR	-0,6781
67	2156	lacR	-0,9260
68	2160	2160	0,0000
69	2216	2216	-0,1375
70	2247	sarY	0,6781
71	2248	2248	-1,5850
72	2264	rpiRC	0,3785
73	2271	hex	0,0000
74	2279	hutR	-0,6781
75	2300	2300	0,3785
76	2303	tcaR	0,1375
77	2308	hssR	0,2630
78	2310	2310	-0,6781
79	2322	2322	0,8480
80	2326	rsp	-0,3785
81	2331	sarZ	0,6781
82	2336	2336	0,0000
83	2337	nrec	0,2630
84	2437	sarT	-0,8480
85	2438	sarU	-0,6781
86	2445	2445	-1,0704
87	2459	2459	-0,8480
88	2480	cidR	-0,3785
89	2490	2490	0,2630
90	2509	2509	-1,0000
91	2515	2515	0,0000
92	2530	2530	0,1375
93	2547	2547	-0,8480
94	2559	nsaR	0,8480
95	2566	arcR	-0,1375
96	2571	argR3	-0,1375
97	2575	2575	0,0000
98	2599	icaR	0,4854
99	2640	2640	0,1375

The following transcriptional regulator mutants were excluded from analysis, because they did not grow in SSM within 72 h

100	444	gtlC
101	450	treR
102	605	sarA
103	1174	1174

The following transcriptional regulator mutants were not available at time of acquisition

1	804	804
2	1798	airR
3	1888	hisR
4	2218	sarV
5	2425	2425

https://doi.org/10.1098/rspb.2026.1930

Table S7

S. aureus Nebraska Transposon Mutant Library (NTML)

The Nebraska Transposon Mutant Library (NTML) provides a comprehensive collection of knockouts of specific genes from a wild-type strain, including knockouts of transcriptional regulators, via transposon mutagenesis (Fey et al., 2013). The wild-type strain JE2 is a USA300 plasmid-cured derivative (USA300_FPR3757) of strain LAC (Boles, Thoendel, Roth, & Horswill, 2010), a well-characterised community-associated methicillin-resistant *S. aureus* (CA-MRSA) strain isolated from the Los Angeles County jail. Plasmids were cured to facilitate genetic manipulation and avoid interference during transposon mutagenesis experiments, resulting in strain USA300 JE2. A list of 108 NTML strains, where transcriptional regulators are knocked out, is provided in Gimza *et al.* 2019. Of these 108 mutants, 103 were available at the time of acquisition of the NTML.

Comparison with data from Gimza et al.

Gimza *et al.* 2019 screened transposon mutants for all 108 available transcriptional regulators in *S. aureus* USA300 JE2 from the Nebraska Transposon Mutant Library (NTML) and reported the alterations in proteolytic activity using gelatin zymography normalised to the band intensity of the USA300 JE2 wild-type strain. We extracted the fold change in band intensity from their Fig. 5, transformed it to the $\log_2$ fold ratio between mutant and wild-type, and performed a pairwise Pearson correlation analysis of the proteolytic capacity and adaptation time τ, and of the proteolytic capacity and maximum growth rate ρ_{max} (Supplementary Fig. S4C and D).

https://doi.org/10.1098/rspb.2026.1930

Table S8.

The oligonucleotides employed for genetic manipulation in this research

Primer name	5'-3' sequence	Description
OL7735	ATGggatccGAGCAGGTGCGAAATAATGG	*sigB* confirmation F
OL7736	ATGgaattcCAAATTCTATTGATGTGCTGCTTCTTG	*sigB* confirmation R
aur A	cctcactaaagggaacaaaagctgggtaccCATTAGGCATCTGGTTTGTC	*aur* deletion construct generation in pIMAY-Z
aur B	gtttaacattacttcttcttgtttatTTCCTCCTGAAATCTTAAAAACAG	*aur* deletion construct generation in pIMAY-Z
aur C	ctgtttttaagatttcaggaggaaATAAACAAGAAGAAGTAATGTTAAAC	*aur* deletion construct generation in pIMAY-Z
aur D	cgactcactatagggcgaattggagctcTATGAACCATTGATGATTGAACT	*aur* deletion construct generation in pIMAY-Z
aur OUT F	AACGCGATTAAGTATGAT	Screen for *aur* deletion mutants
aur OUT R	CCAGGTGAGGTTTTGAC	Screen for *aur* deletion mutants
sspA A	cctcactaaagggaacaaaagctgggtaccGGACGTCGTGAACTA	*sspA* deletion construct generation in pIMAY-Z
sspA B	tactaaatctaaattaagatgaagtttaCATCTAAAAACCTCCAAAAAA	*sspA* deletion construct generation in pIMAY-Z
sspA C	ttttttggaggtttttagatgTAAACTTCATCTTAATTTAGATTTAGTA	*sspA* deletion construct generation in pIMAY-Z
sspA D	attggagctccaccgcggtggcggccgcTTTAGCACTTTCTTTTTCTTTTACA	*sspA* deletion construct generation in pIMAY-Z
sspA OUT F	GCAATCGTTCCAGGCTCATC	Screen for *sspA* deletion mutants
sspA OUT R	TCTTCTTGTATCGCTTCGTTTTC	Screen for *sspA* deletion mutants
pIMAY-Z MCS F	TACATGTCAAGAATAAACTGCCAAAGC	Screen pIMAY-Z constructs
pIMAY-Z MCS R	AATACCTGTGACGGAAGATCACTTCG	Screen pIMAY-Z constructs
rsbV _Trunc_F	AGGGAACAAAAGCTGGGTACCTTAAAAAACATAAACATATGCACC	Repair 61-bp truncation in *rsbV*
rsbV _Trunc_R	ATAGGGCGAATTGGAGCTCTTATATACTTAGTCATACTGATTGTC	Repair 61-bp truncation in *rsbV*

https://doi.org/10.1098/rspb.2026.1930

Supplementary Method M1

Calculation of the parameters adaptation time τ and maximum (absolute) growth rate ρ_{max}

Measuring bacterial density and growth patterns using optical density (OD) is fast and reliable. However, the medium may in itself absorb light and, since the amount of absorbed light differs between media, the absolute bacterial density cannot measured. Values are instead normalised by subtracting the background OD, so that each measurement series starts at approximately zero. At later time points, equating the normalised OD to the bacterial density may be justified since the deviations from zero at the start become negligible, but that does not apply to the initial period of slow growth. Hence, only the absolute growth rate DX/Dt, where X is the bacterial concentration and t the time, can be calculated from the data as DOD/Dt for the entire series, rather than the relative or specific growth rate (DX/X)/Dt. (Note that using derivatives instead of differences between subsequent measurements for these calculations requires fitting of differentiable curves to the data. As the shape of our growth curves varies widely, such fitting would require further, perhaps questionable, assumptions.) We therefore measure bacterial densities at OD_{600} and calculate the parameters "adaptation time" τ and "maximum growth rate" ρ_{max} from OD_{600} time series data as follows (Fig. 1B): From the empirical curve (red) differences of OD_{600} between neighbouring time points are calculated; the maximum difference corresponds to the maximum growth rate ρ_{max} (in units of ΔOD_{600}/h). The line thus defined is extended to the intersection with the x-axis. The length of time from the start of the experiment to this intersection is taken as the adaptation time τ (in units of h). Furthermore, we note that measurements of bacterial concentrations via OD are only linear for low bacterial concentrations. In milk, an additional problem is clotting and curd formation at higher bacterial concentrations. We therefore only consider and show bacterial concentrations in SSM up to OD_{600} = 1. In fresh milk with 3.5% fat content, the problem is exacerbated: while the growth characteristics of TG13a (SigB-wt), TG13o and TG13aR (both SigB-def) are initially similar to those in SSM, increased clotting and curd formation compromise the measurements from about OD_{600} = 0.1, i.e., after about 4h in TG13o and TG13aR and 10h in TG13a (Supplementary Fig. S1G).

Supplementary Method M2

***Asp23* expression (RT-qPCR)**

Asp23 expression was determined at the following time points: In inoculum (TSB, 18 - 20h) for TG13a, TG13o, and TG13aR; in SSM after 2 and 4h for TG13a; in SSM after 8h for TG13a, TG13o and TG13aR; and in SSM after 16, 20 and 24h for TG13a. Depending on the actual bacterial density, batch cultures with either 15 or 50ml of SSM were utilised to obtain adequate quantities of bacteria.

Supplementary Method M3

Handling Poorly Growing Strains in Statistical Analyses

Some human strains hardly grew in SSM (at least during the study period), so their adaptation time and maximal growth rate could not be calculated reliably. Leaving these strains out of statistical analyses generally decreased differences between human and

https://doi.org/10.1098/rspb.2026.1930

bovine strains and lowered the sample size, thus resulting in conservative tests. Therefore, we opted for this approach. Note that the cases where some strains did not grow at all are mentioned in the main text or figures wherever they occurred.

https://doi.org/10.1098/rspb.2026.1930

References

Antók, F. I., Mayrhofer, R., Marbach, H., Masengesho, J. C., Keinprecht, H., Nyirimbuga, V., . . . Loncaric, I. (2019). Characterization of Antibiotic and Biocide Resistance Genes and Virulence Factors of Staphylococcus Species Associated with Bovine Mastitis in Rwanda. *Antibiotics (Basel), 9*(1). doi:10.3390/antibiotics9010001

Boles, B. R., Thoendel, M., Roth, A. J., & Horswill, A. R. (2010). Identification of genes involved in polysaccharide-independent *Staphylococcus aureus* biofilm formation. *PLoS One, 5*(4), e10146. doi:10.1371/journal.pone.0010146

Chan P.F., Foster S.J., Ingham E., Clements M.O. 1998 The *Staphylococcus aureus* alternative sigma factor sigmaB controls the environmental stress response but not starvation survival or pathogenicity in a mouse abscess model. *J Bacteriol.* 180(23), 6082-6089. (doi:10.1128/jb.180.23.6082-6089.1998).

Duthie, E. S., & Lorenz, L. L. (1952). Staphylococcal coagulase; mode of action and antigenicity. *J Gen Microbiol, 6*(1-2), 95-107. doi:10.1099/00221287-6-1-2-95

Dyke, K. G., Jevons, M. P., & Parker, M. T. (1966). Penicillinase production and intrinsic resistance to penicillins in Staphylococcus aures. *Lancet, 1*(7442), 835-838. doi:10.1016/s0140-6736(66)90182-6

Fey, P. D., Endres, J. L., Yajjala, V. K., Widhelm, T. J., Boissy, R. J., Bose, J. L., & Bayles, K. W. (2013). A Genetic Resource for Rapid and Comprehensive Phenotype Screening of Nonessential *Staphylococcus aureus* Genes. *MBio, 4*(1). doi:10.1128/mBio.00537-12

Fitzgerald, J. R., Hartigan, P. J., Meaney, W. J., & Smyth, C. J. (2000). Molecular population and virulence factor analysis of *Staphylococcus aureus* from bovine intramammary infection. *J Appl Microbiol, 88*(6), 1028-1037.

Grunert, T., Stessl, B., Wolf, F., Sordelli, D. O., Buzzola, F. R., & Ehling-Schulz, M. (2018). Distinct phenotypic traits of *Staphylococcus aureus* are associated with persistent, contagious bovine intramammary infections. *Sci Rep, 8*(1), 15968-15968. doi:10.1038/s41598-018-34371-1

Grunert, T., Wenning, M., Barbagelata, M. S., Fricker, M., Sordelli, D. O., Buzzola, F. R., & Ehling-Schulz, M. (2013). Rapid and reliable identification of *Staphylococcus aureus* capsular serotypes by means of artificial neural network-assisted Fourier transform infrared spectroscopy. *J Clin Microbiol, 51*(7), 2261-2266. doi:10.1128/JCM.00581-13

Horsburgh, M. J., Aish, J. L., White, I. J., Shaw, L., Lithgow, J. K., & Foster, S. J. (2002). sigmaB modulates virulence determinant expression and stress resistance: characterization of a functional rsbU strain derived from *Staphylococcus aureus* 8325-4. *J Bacteriol, 184*(19), 5457-5467. doi:10.1128/jb.184.19.5457-5467.2002

Johler, S., Stephan, R., Althaus, D., Ehling-Schulz, M., & Grunert, T. (2016). High-resolution subtyping of *Staphylococcus aureus* strains by means of Fourier-transform infrared spectroscopy. *Syst Appl Microbiol*. doi:10.1016/j.syapm.2016.03.003

Keane, O. M., Budd, K. E., Flynn, J., & McCoy, F. (2013). Pathogen profile of clinical mastitis in Irish milk-recording herds reveals a complex aetiology. *Vet Rec, 173*(1), 17. doi:10.1136/vr.101308

Kummel, J., Stessl, B., Gonano, M., Walcher, G., Bereuter, O., Fricker, M., . . . Ehling-Schulz, M. (2016). *Staphylococcus aureus* Entrance into the Dairy Chain: Tracking S. aureus from Dairy Cow to Cheese. *Front Microbiol, 7*, 1603. doi:10.3389/fmicb.2016.01603

Marbach, H., Mayer, K., Vogl, C., Lee, J. Y. H., Monk, I. R., Sordelli, D. O., . . . Grunert, T. (2019). Within-host evolution of bovine *Staphylococcus aureus* selects for a SigB-deficient pathotype characterized by reduced virulence but enhanced proteolytic activity and biofilm formation. *Sci Rep, 9*(1), 13479. doi:10.1038/s41598-019-49981-6

Monk I.R., Shah I.M., Xu M., Tan M.-W., Foster T.J. 2012 Transforming the Untransformable: Application of Direct Transformation To Manipulate Genetically *Staphylococcus aureus* and *Staphylococcus epidermidis*. *mBio* 3(2), 10.1128/mbio.00277-00211. (doi:doi:10.1128/mbio.00277-11).

Murphy, M. P., Niedziela, D. A., Leonard, F. C., & Keane, O. M. (2019). The in vitro host cell immune response to bovine-adapted *Staphylococcus aureus* varies according to bacterial lineage. *Sci Rep, 9*(1), 6134. doi:10.1038/s41598-019-42424-2

Niedziela, D. A., Cormican, P., Foucras, G., Leonard, F. C., & Keane, O. M. (2021). Bovine milk somatic cell transcriptomic response to *Staphylococcus aureus* is dependent on strain genotype. *BMC Genomics, 22*(1), 796. doi:10.1186/s12864-021-08135-7

Niedziela, D. A., Murphy, M. P., Grant, J., Keane, O. M., & Leonard, F. C. (2020). Clinical presentation and immune characteristics in first-lactation Holstein-Friesian cows following intramammary infection with genotypically distinct *Staphylococcus aureus* strains. *J Dairy Sci, 103*(9), 8453-8466. doi:10.3168/jds.2019-17433

Novick, R. (1967). Properties of a cryptic high-frequency transducing phage in *Staphylococcus aureus*. *Virology, 33*(1), 155-166. doi:10.1016/0042-6822(67)90105-5

https://doi.org/10.1098/rspb.2026.1930

Prasad, L. B., & Newbould, F. H. (1968). Inoculation of the bovine teat duct with Staph. Aureus: the relationship of teat duct length, milk yield and milking rate to development of intramammary infection. *Can Vet J, 9*(5), 107-115.

Schabauer, A., Pinior, B., Gruber, C. M., Firth, C. L., Käsbohrer, A., Wagner, M., . . . Obritzhauser, W. (2018). The relationship between clinical signs and microbiological species, spa type, and antimicrobial resistance in bovine mastitis cases in Austria. *Vet Microbiol, 227*, 52-60. doi:10.1016/j.vetmic.2018.10.024

Tuchscherr, L., Bischoff, M., Lattar, S. M., Noto Llana, M., Pförtner, H., Niemann, S., . . . Löffler, B. (2015). Sigma Factor SigB Is Crucial to Mediate *Staphylococcus aureus* Adaptation during Chronic Infections. *PLoS Pathog, 11*(4), e1004870. doi:10.1371/journal.ppat.1004870

Tuchscherr, L. P., Gomez, M. I., Buzzola, F. R., Calvinho, L. F., Lee, J. C., & Sordelli, D. O. (2007). Characterization of a new variant of IS257 that has displaced the capsule genes within bovine isolates of *Staphylococcus aureus*. *Infect Immun, 75*(11), 5483-5488. doi:10.1128/IAI.00747-07

Voyich, J. M., Braughton, K. R., Sturdevant, D. E., Whitney, A. R., Said-Salim, B., Porcella, S. F., . . . DeLeo, F. R. (2005). Insights into Mechanisms Used by *Staphylococcus aureus* to Avoid Destruction by Human Neutrophils. *J Immunol, 175*(6), 3907-3919.

Walzl, A., Marbach, H., Belikova, D., Vogl, C., Ehling-Schulz, M., Heilbronner, S., & Grunert, T. (2023). Prevalence of the SigB-Deficient Phenotype among Clinical *Staphylococcus aureus* Isolates Linked to Bovine Mastitis. *Antibiotics, 12*(4), 699.

Wörmann, M. E., Reichmann, N. T., Malone, C. L., Horswill, A. R., & Gründling, A. (2011). Proteolytic cleavage inactivates the *Staphylococcus aureus* lipoteichoic acid synthase. *J Bacteriol, 193*(19), 5279-5291. doi:10.1128/jb.00369-11

https://doi.org/10.1098/rspb.2026.1930
