# Supplementary material for: A single transcriptional regulator is crucial for the adaptation of Staphylococcus aureus to diverse niches
Source: Proc Biol Sci. 2026 Oct 14;293(2080):20261930. doi: 10.1098/rspb.2026.1930 (PMC13678726; doi:10.1098/rspb.2026.1930)
Supplement: Supplementary file 1 [file rspb.2026.1930.zip › rspb.2026.1930/rspb20261930_si_002.docx]

**Supplementary material for**

**Proceedings of the Royal Society B: Biological Sciences** (**Proc. R. Soc. B)**

**Supplementary Figures**

**Fig. S1.**

**Title:** Comparison of TG13a (SigB-wt), and TG13o and TG13aR (both SigB-def)

**Caption: (A)** *S. aureus* strains pre-cultivated in TSB and then transferred to fresh TSB grow without an adaptation phase. **(B)** Population density in CFU/ml after transfer to SSM. The arrows mark the time of curd formation. **(C)** Competition experiment TG13o vs TG13a. Boxplots of the proportion of the derived SigB-deficient strain TG13o after 24, 48, 72 and 86 h in SSM and TSB, depending on the proportion of TG13o (vs TG13a) in the initial inoculum. We test if the *in vivo* evolved SigB-def strain (TG13o) has a growth advantage over the initially dominant SigB-wt strain (TG13a) after transfer from TSB to SSM by co-cultivating the strains from different starting proportions of the inoculum (0.1, 0.5, and 0.9 of TG13o). Concurrently, a control experiment involving transfer of the same inocula to fresh TSB is performed. After 24, 48, 72 and 96 h, yellow and white CFUs are counted to determine the proportion of TG13o. In total, four independent iterations of this experiment are conducted, two for early (24 and 48 h) and two for late time points (72 and 96 h). (**D**) Relative *asp23* mRNA expression (RT-qPCR; mean ± SD) in the interval (h) after transfer from TSB to SSM. Differences in pairwise means were tested with Tukey’s HSD. The overlapping range of SigB activity in SigB-wt and SigB-def strains is underlaid. **(E)** Transmission Electron Microscopy (TEM) images of SSM without bacteria (media control), and after growth for 24 h in SSM. For TEM, bacterial and control samples were fixed, dehydrated, and embedded in resin before being cut into ultrathin sections and stained. The prepared sections were then examined using a Zeiss TEM 900 microscope operated at 50 kV. **(F)** Sodium dodecyl sulfate(SDS)-polyacrylamide gel electrophoresis and silver staining were performed to visualise the SSM degradation pattern induced by TG13a (SigB-wt) and TG13o/TG13aR (SigB-def) using an isocratic 15%T, 18 cm x 16 cm separation gel. Media controls without bacteria, labelled C, are included at time points t = 0, t = 8 and t =24. Sample load was normalised to equal protein concentration. **(G)** The growth curves (OD600) of TG13a (SigB-wt), TG13o, and TG13aR (both SigB-def) after transfer from TSB to fresh milk with 3.5% fat content (OD600; mean – SD).

M, Marker proteins. TG13a, SigB-wt, TG13o and TG13aR, both SigB-def.

**Fig. S2.**

**Title:** In-host evolution towards SigB-deficiency in bovine mastitis isolates of various clonal complexes

**Caption: (A)** Intervals between the first and the last isolate collected from individual dairy cows (n = 20), and **(B)** number of isolates collected from each cow (from which a minimum of three isolates was obtained) by retrospective screening for SigB-def identifiable by visible loss of colony pigmentation (i.e., for a white or grey instead of a yellow colony colour) from consecutively sampled isolates of *S. aureus* from bovine hosts with persistent IMI (n = 20). **(C)** Putative SigB-def isolates in milk samples from three cows from two herds. Minimum spanning tree using core genome multilocus sequence typing (cgMLST) data for TG22 and TG11/TG12 (same herd) suggests clonality, although the isolates were recovered from different cows, suggesting transmission from one cow to the other. We used a cluster threshold of ≤24 allelic differences in the core genome to determine clonality. For TG22, the first and last isolates were collected 15 months apart. Both of these, as well as the three intermediate isolates collected from the same cow, have a cgMLST cluster of less than 24 allelic differences, indicating clonality. Isolates were assigned to the clonal complexes CC97, CC151, and CC1. **(D)** Isolate sampling and SigB activity: In both reconstructed strains (TG22jR and TG12fR) carotenoid pigmentation is restored, confirming that the mutations are solely responsible for the inactivation of the *sigB* operon.

**Fig. S3.**

**Title:** Genetic relatedness of bovine-associated CC97 and CC151 isolates

**Caption:** Minimum spanning tree using core genome multilocus sequence typing (cgMLST) data for strains of **(A)** CC97 (n = 13) and **(B)** CC151 (n = 13) isolated from the milk of cows from different geographic regions (see the list of strains and their characteristics in Supplementary Table 4). Graphical representation of their relationships obtained by a cgMLST analysis. All isolates show unique cgMLST patterns, exceeding the threshold of ≤24 allelic differences in the core genome used to determine clonality. The colour of the nodes indicates the ST type.

**Fig. S4.**

**Title:** Screening of NTML regulatory mutant strains

**Caption:** Means and standard errors of the differences between each NTML mutant strain and the wild-type reference strain with respect to **(A)** adaptation time (*τ*) and **(B)** maximal growth rate (*ρ*max). The pink colour indicates significant differences as determined by Dunnett's test with the wild-type as a reference. The four mutants *gltC*, *treR*, *sarA,* and NE40 (SAUSA300_1174) were excluded from the analysis because they did not grow in SSM within 72 h. For each NTML regulatory mutant strain, the difference in proteolytic capacity from the reference JE2 strain (on the x-axis) is plotted **(C)** vs the difference in the ln-transformed adaptation time (*τ*), and **(D)** the maximal growth rate (*ρ*max) (y-axis) from the reference JE2 strain. The pairwise Pearson correlations are not significant and therefore not shown.

**Supplementary Tables**

**Table S1.**

**Title:** Bovine-associated isolates from persistent IMI and engineered mutants

**Caption:** The isolates TG13a (SigB-wt) and TG13o (SigB-def) were described in our previous study (Marbach et al., 2019). The single nucleotide change *rsbU*(G368A) observed in TG13o was introduced into TG13a, and the resulting strain designated as TG13aR (Walzl et al., 2023). In addition, we engineered isogenic knockout mutants of the proteases Aur (Δ*aur*) and SspA (Δ*sspA*) based on the SigB-wt (TG13a) and SigB-def strains (TG13o/ TG13aR). The thermosensitive plasmid pIMAY-Z was utilised to generate *aur* and *sspA* deletion mutants using previously described protocols (Monk et al., 2012). Briefly, oligonucleotide primers were used to amplify ~500 bp AB and CD flanking regions of each gene of interest (Supplementary Table S8) using Q5 polymerase (NEB). Gibson assembly (NEB) generated pIMAY-Z constructs that were transformed firstly into *E. coli* DC10B (Monk et al., 2012) and then, after Sanger sequencing confirmation (Eurofins), into the *S. aureus* strain of interest. After integration at 37°C and plasmid excision at 30°, OUT PCR was used to confirm that the clones contained gene deletions.

The isolates TG11-CC1 (n = 3), TG12-CC1 (n = 3) and TG22-CC151 (n = 5) were obtained by retrospectively screening 105 staphylococcal isolates from the milk of 20 cows with persistent IMI for visible loss of colony pigmentation (i.e., for a white or grey instead of a yellow colour). Note that we consider IMI persistent when clonal isolates can be collected from at least three consecutive milk samples from the same dairy cow, with a minimum of one month between the first and the last of these three samples. This screen yielded putative SigB-def isolates in milk samples from three cows from two herds. The isolates TG22-CC151 were from one udder quarter of a single cow (cow 11 in study Grunert, et al. 2018): Isolates from samples taken within the first two weeks (TG22a and d) are SigB-wt, while those from samples taken in weeks 33 (TG22g), 52 (TG22i), and 64 (TG22j) are SigB-def. The isolates TG11-CC1 (n = 3) and TG12-CC1 (n = 3) came from cows of the same dairy herd in Austria, and were collected from milk samples taken during routine microbial mastitis diagnostic screening over 15 weeks. In fact, TG11-CC1 and TG12-CC1 isolates were collected at weeks 1, 4 and 15 from two different cows. While TG11a and TG11b are SigB-wt, a stop codon in the *sigB* gene of TG11c causes SigB-deficiency. All three TG12 isolates (a, e, and f) are SigB-def due to the SNP G431T in *rsbU*. Genome sequencing of these isolates reveals clonality within isolates from the same cow, indicating persistent infection with the same strain. Although the isolates TG11 and TG12 were recovered from different cows, genome sequencing indicates clonality, suggesting transmission from one cow to the other. We also engineered the following isogenic mutant strains: In strain TG12fR we reversed the point mutation *rsbU*:431G→T (Walzl et al., 2023). To restore the 62 bp truncation in *rsbV* TG22j, the full-length allele was amplified (Supplementary Table S8) from the precursor isolate TG22a and cloned into pIMAY. Allelic exchange was performed as described above (Monk et al., 2012). The resulting strain was named TG22jR.

**Table S2.**

**Title:** Logistic regression analysis of the competition experiment of TG13o vs TG13a

**Caption:** Inncolum with different proportions of TG13o (0.1, 0.5, 0.9) was grown in both TSB and SSM, and the growth of each strain was evaluated at different time points (24h, 48h, 72h, 96h). The dependent variable in the regression analysis is the log-odds of the observed strain proportions. This is regressed onto the independent variables encoding the medium (TSB corresponds to 0; SSM to 1), the evaluation times, and the log-odds of the initial strain proportions (note this precludes inclusion of an intercept). Including a random effect term for different replicates does not improve model fit (not shown).

**Table S3.**

**Title:** Average difference between TG13a, TG13o, and TG13aR of lactose consumption, production of metabolites, pH, and proteolytic activity after 8 h and 24 h of growth in SSM (*p*-values of t-tests shown)

**Caption:** not required

**Table S4.**

**Title:** Bovine isolates for comparison of CC97 and CC151

**Caption:** We selected the strain pair MOK023-CC97 and MOK124-CC151 because comprehensive *in vivo* and *in vitro* data are available (Murphy, Niedziela, Leonard, & Keane, 2019; Niedziela, Cormican, Foucras, Leonard, & Keane, 2021; Niedziela, Murphy, Grant, Keane, & Leonard, 2020). MOK023-CC97 (ST3170) and MOK124-CC151 (ST151) strains were originally isolated from cases of clinical mastitis (Keane, Budd, Flynn, & McCoy, 2013). The CC97 and CC151 isolates (n = 13, each) used for comparative analyses in this article originate from our collection of well-characterised strains from bovine mastitis milk samples (Anto k et al., 2019; Fitzgerald, Hartigan, Meaney, & Smyth, 2000; Tom Grunert et al., 2018; T. Grunert et al., 2013; Kummel et al., 2016; Prasad & Newbould, 1968; Schabauer et al., 2018; L. P. Tuchscherr et al., 2007). Strains were selected to represent diverse geographic origins (Austria, n = 11; Argentina, n = 9; Rwanda, n = 4; others, n = 2), sequence types (STs), and *spa*-types. Core genome multilocus sequence typing (cgMLST) and subsequent analyses are described in the subsection: Genome sequencing and molecular genetic typing. An overview of cgMLST analyses of all strains is provided in Supplementary Fig. S3.

**Table S5.**

**Title:** Human-associated isolates (CC8) and engineered mutants

**Caption:** Strain 8325-4 is a widely used model strain originally isolated from a sepsis patient in 1960 (strain RN1 or NCTC8325); all three prophages were cured by UV radiation (Novick, 1967). Strain 8325-4 is SigB-def because of an 11-bp deletion in *rsbU*, which was repaired in strain SH1000 (SigB-wt) (Horsburgh et al., 2002). The isogenic mutant SH1000Δ*sigB* (SigB-def) was derived from SH1000 (L. Tuchscherr et al., 2015). Strain Newman (Nm, NCTC8178) is SigB-wt and was isolated from a patient with osteomyelitis (Duthie & Lorenz, 1952). We introduced the same single nucleotide change in *rsbU*(G368A) that we studied in TG13o to the Newman strain to make the SigB-def mutant strain NmR (Walzl et al., 2023). The SigB-wt strain LAC (CA-MRSA USA300) was originally isolated from a human skin and soft tissue infection (Voyich et al., 2005), and we engineered the isogenic mutant LACΔ*sigB* (SigB-def) from it. In addition, we combined a derivative of the LAC strain that lacks all 10 major secreted proteases (LAC*protease KO) with a SigB-def mutant, LAC* protease KOΔ*sigB*. The protease null strain (Δ*aur*Δ*sspAB*Δ*scpAspl*::erm) was originally generated by (Wo rmann, Reichmann, Malone, Horswill, & Gru ndling, 2011) and the originating *sigB*::tet mutation was generated by Chan et al., 1998. Strains were constructed using Φ11 phage lysates containing *sigB*::tet and transduced into either the USA300 LAC background (Boles, Thoendel, Roth, & Horswill, 2010), or the USA300 LAC protease null background. Transduced strains were selected on TSA containing 5 μg/mL tetracycline and confirmed via PCR using the primers OL7735 and OL7736.

**Table S6.**

**Title:** *S. aureus* isolates for comparison of human- and bovine-associated CC

**Caption:** A set of 13 human- and 10 bovine-associated isolates was used. The subset of human isolates includes major MRSA and MSSA epidemic clones, specifically: Four isolates of CC8, and three isolates of CC30, CC5, and CC45 each. These represent CCs commonly isolated from asymptomatic and infected humans (Duthie & Lorenz, 1952; Dyke, Jevons, & Parker, 1966; Johler, Stephan, Althaus, Ehling-Schulz, & Grunert, 2016). The subset of bovine-associated isolates comprises three strains each of CC97 and CC151, and four isolates of bovine CC8 (Fitzgerald et al., 2000; Kummel et al., 2016; Prasad & Newbould, 1968; Schabauer et al., 2018; L. P. Tuchscherr et al., 2007). Within each CC, strains were selected to represent diverse sequence types (ST, MLST) and *spa*-types.

**Table S7.**

**Title:** *S. aureus* Nebraska Transposon Mutant Library (NTML)

**Caption:** The Nebraska Transposon Mutant Library (NTML) provides a comprehensive collection of knockouts of specific genes from a wild-type strain, including knockouts of transcriptional regulators, via transposon mutagenesis (Fey et al., 2013). The wild-type strain JE2 is a USA300 plasmid-cured derivative (USA300_FPR3757) of strain LAC (Boles, Thoendel, Roth, & Horswill, 2010), a well-characterised community-associated methicillin-resistant *S. aureus* (CA-MRSA) strain isolated from the Los Angeles County jail. Plasmids were cured to facilitate genetic manipulation and avoid interference during transposon mutagenesis experiments, resulting in strain USA300 JE2. A list of 108 NTML strains, where transcriptional regulators are knocked out, is provided in Gimza *et al. 2019.* Of these 108 mutants, 103 were available at the time of acquisition of the NTML.

**Comparison with data from Gimza et al.**

Gimza *et al.* 2019 screened transposon mutants for all 108 available transcriptional regulators in *S. aureus* USA300 JE2 from the Nebraska Transposon Mutant Library (NTML) and reported the alterations in proteolytic activity using gelatin zymography normalised to the band intensity of the USA300 JE2 wild-type strain. We extracted the fold change in band intensity from their Fig. 5, transformed it to the log2 fold ratio between mutant and wild-type, and performed a pairwise Pearson correlation analysis of the proteolytic capacity and adaptation time (*τ*), and of the proteolytic capacity and maximum growth rate *ρ*_max_ (Supplementary Fig. S4C and D).

**Table S8.**

**Title:** The oligonucleotides employed for genetic manipulation in this research

**Caption:** not required

**Supplementary Methods**

**Supplementary Method M1**

**Title:** Calculation of the parameters adaptation time *τ* and maximum (absolute) growth rate *ρ*_max_

**Caption:** not required

**Supplementary Method M2**

**Title:** *Asp23* expression (RT-qPCR)

**Caption:** not required

**Supplementary Method M3**

**Title:** Handling Poorly Growing Strains in Statistical Analyses

**Caption:** not required
